# *Oxygels*: Tunable Phosphorescent Hydrogels for Cherenkov‐Excited Luminescence Imaging (CELI) of Oxygen

**DOI:** 10.1002/adhm.71586

**Published:** 2026-08-12

**Authors:** Simin Belali, Marien Iliza Ochoa Mendoza, Matthew S. Reed, Danylo Mykhalchuk, Annemarie Lang, Joel D. Boerckel, Brian W. Pogue, Sergei A. Vinogradov

**Affiliations:** ^1^ Department of Biochemistry and Biophysics Perelman School of Medicine University of Pennsylvania Philadelphia PA USA; ^2^ Department of Chemistry School of Arts and Sciences University of Pennsylvania Philadelphia PA USA; ^3^ Department of Medical Physics School of Medicine University of Wisconsin‐Madison Madison Wisconsin USA; ^4^ Thayer School of Engineering Dartmouth College Hanover NH USA; ^5^ Department of Orthopaedic Surgery Perelman School of Medicine University of Pennsylvania Philadelphia PA USA; ^6^ Department of Orthopaedic Surgery University of Michigan Medical School Ann Arbor Michigan USA; ^7^ Department of Bioengineering School of Engineering and Applied Science University of Pennsylvania Philadelphia PA USA

**Keywords:** Cherenkov‐excited luminescence, hydrogel, oxygen, Oxyphor, phosphorescence, porphyrin

## Abstract

We present a family of phosphorescent hydrogels, termed *Oxygels*, for local deep‐tissue oxygen sensing by Cherenkov‐excited luminescence imaging (CELI). *Oxygels* comprise dendritic phosphorescent probes covalently bound within a polyethyleneglycol (PEG) matrix held together by an optically neutral dendritic cross‐linker. Covalent attachment of the phosphorescent probe within the matrix prevents its leaching from the gel. Changing the size/generation of the dendrimer surrounding the phosphorescent center enables tuning of *Oxygel* sensitivity, while varying the ratio between the probe and neutral cross‐linker helps to optimize *Oxygel* performance. Upon implantation into tissue, *Oxygels* induced only a moderate initial foreign body response with minimal macrophage recruitment. The performance of the *Oxygels* in CELI was evaluated using electron pulses from a linear accelerator (linac) for Cherenkov excitation. Robust phosphorescence decays were detected from *Oxygel*s implanted subcutaneously or abdominally in mice in vivo using peripheral detectors upon excitation by clinical radiation doses (up to 10 Gy) delivered in FLASH mode. The resulting decay time constants provide quantitative information about tissue oxygenation at the site of *Oxygel* implantation.

## Graphical abstract

Biocompatible hydrogels with covalently embedded near‐infrared phosphorescent probes enable local tissue pO_2_ quantification by means of Cherenkov‐excited luminescence imaging (CELI), where optical excitation occurs within tissues upon irradiation with high‐energy electron beams.

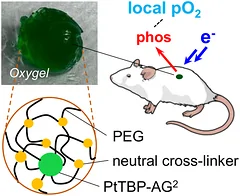

## 1 Introduction

Oxygen is a key metabolite, whose average concentration in tissue at rest and/or at elevated metabolic loads carries information about the tissue physiological status, potentially serving as a marker of disease [1, 2]. Oxygen also plays a critical role in some types of tissue therapy, such as radiation therapy (RT) and photodynamic therapy, where the ability to noninvasively follow changes in tissue partial pressure of oxygen (pO_2_) would be extremely valuable for monitoring progress of such treatments, both from the physiological point of view, to keep track of the tissue metabolic status, and from the therapeutic perspective, to ensure that therapy is administered with maximal efficiency.

A prominent approach to biological sensing of oxygen relies on its ability to quench molecular phosphorescence [3, 4]. In the past, the phosphorescence quenching method has been implemented in a number of ways, ranging from measurements in cell cultures, to tissue fiber optic oximetry, to different forms of microscopic and macroscopic imaging; and numerous soluble and polymer‐supported phosphorescent probes have been developed [5, 6, 7, 8]. For microscopy applications and imaging at tissue surface probes with optical bands in the UV/visible range can be used; however, for deeper imaging, excitation and emission in the red/near‐infrared are required, since light at shorter wavelengths is scattered and strongly absorbed by endogenous chromophores, limiting sensing to just a few tens of microns under the tissue surface. Using red‐absorbing/near‐infrared emitting phosphorescent probes [9, 10, 11, 12, 13], oxygen imaging is possible through centimeters of tissue [13], which has been exploited in the development of phosphorescence lifetime tomography [14, 15]. The latter method enables visualization of volumetric oxygen distributions in tissue in 3D; however, like all all‐optical tomographic techniques, it is plagued by the problem of low spatial resolution, caused by strong scattering of optical photons in tissue.

To overcome the scattering problem, we have developed a mixed modality method, termed Cherenkov‐excited luminescence imaging (CELI), for sensing oxygen and potentially other analytes in the setting of clinical RT [16, 17, 18, 19, 20]. In the CELI technique, optical probes are excited not by light from external sources, but by Cherenkov light [21] generated internally within tissue upon application of high‐energy particle beams [18, 22]. X‐rays and accelerated electrons are scattered in tissue much less than optical photons, while Cherenkov radiation is produced strictly along the particles' tracks, which ultimately serves to reduce the uncertainty in predicting the excitation path and helps to improve imaging resolution. For example, by scanning a radiation beam shaped like a thin sheet across an object and recording the emission from each dissected plane, tomographic images of oxygen can be obtained with sub‐millimeter resolution in a variant of the method termed Cherenkov‐excited luminescence scanned imaging (CELSI) [23].

In most cases, CELI (or CELSI) of oxygen has been performed using soluble oxygen probes, Oxyphors [5, 12, 13, 20]. When delivered systematically, Oxyphors permit tomographic imaging of large tissue volumes; however, for practical clinical applications, it could be useful to measure oxygen in a specific location(s) rapidly and longitudinally, for example, in tumors undergoing RT over a course of days/weeks, rather than to perform long data acquisitions for complete 3D image reconstructions. For that, Oxyphors can be injected directly (extravascularly) into tissue [24] and serve as local oxygen sensors. However, being highly hydrophilic molecules, they rapidly diffuse away from the injection site and eventually spread throughout the body, while the signal emitted from the site of interest decreases. To that end, phosphorescent probes embedded into hydrogels with appropriately tuned oxygen sensitivity could present a viable alternative to Oxyphors, making up a useful tool for longitudinal local deep tissue oxygen sensing by CELI.

Hydrogels and hydrogel‐like materials have been at the center of attention in the field of biomolecular engineering due to the multitude of their potential applications, ranging from the development of artificial organs to wound sealing to sensing technology [25, 26, 27, 28, 29]. A few phosphorescent polyethyleneglycol (PEG)‐based hydrogels with embedded phosphorescent Pd porphyrins have been developed specifically for oxygen sensing, including in tissue [30], in live cell‐supporting scaffolds [31], as surface coatings for monitoring oxygen tension in wounds [32, 33] and in flow cells [34]. Phosphorescent hydrogels based on other types of polymers have also been reported [35, 36, 37], as well as hydrogels holding non‐covalently embedded phosphorescent oxygen‐sensitive particles [38, 39]. However, with rare exceptions [40, 41], most of the reported phosphorescent hydrogels and related materials [42] feature chromophores whose excitation bands and phosphorescence occur in the visible range, which is appropriate for applications where the optical path is short and/or the samples are essentially transparent, such as in cellularized scaffolds in vitro [31]; or for superficial sensing, where hydrogels are positioned (or “painted”) on the tissue surface, so that the excitation/emission path is unobstructed by tissue itself [32, 33].

In an earlier study, we demonstrated that phosphorescent hydrogels indeed could operate as sensors for Cherenkov‐based sensing [43]. However, the material tested was a standard agarose gel, to which a soluble probe Oxyphor PtG4 [12, 13] was admixed prior to gelation. Upon delivery into tissue, the probe was initially retained in the gel; however, within hours Oxyphor leached from the agarose matrix, precluding longitudinal measurements. To address this limitation as well as limitations of other hydrogel‐based oxygen sensors developed previously, here we report a new family of phosphorescent hydrogels, termed *Oxygels*, designed for deep‐tissue oxygen sensing in the context of CELI. The key features of the new *Oxygels* are their bright optical transitions in the near‐infrared spectral region, biocompatibility and biodegradability, and most importantly, an optimized sensitivity range for accurate oxygen quantification in tissue. The new *Oxygels* were photophysically characterized, calibrated, and tested in CELI in mice, proving that they can serve as efficient implantable local sensors for longitudinal oxygen imaging.

## 2 Experimental Methods

The details of the synthesis and characterization of *Oxygels*, photophysical measurements, phosphorescence lifetime/oxygen calibrations, and the setup for CELI are described in the Supporting Information.

### 2.1 Statistical Analysis

For phosphorescence lifetimes and Stern–Volmer phosphorescence oxygen quenching constants, all measurements were performed in triplicate. The accuracy of lifetime determination is a function on the signal‐to‐noise ratio (SNR) in the phosphorescence data, which, in turn, is dependent on the number of phosphorescence decays (*N*) averaged per single data point as well as on the intrinsic instrumentation parameters. The SNR was defined as the area under the decay curve divided by the average amplitude of noise at the decay tail. In a typical experiment, *N* was 200, and SNR was 200–300, which resulted in accuracy in the decay time constant measurements of ±0.2 µs (SD). Analysis of the Stern–Volmer plots was performed using Origin 7.1 software.

### 2.2 Ethics Statement

All animal procedures were conducted in accordance with the University of Pennsylvania IACUC regulations (U. Penn site; protocol #: 806482) and/or in accordance with the University of Wisconsin IACUC regulations (U. Wisconsin site; protocol #: M006554‐A03).

At the U. Penn site, 16–20 weeks‐old male mice (C57BL/6J, Jackson Laboratory) were housed in pairs in standard plastic ages filled with wooden chips in a conventional (semi‐barrier) animal facility. Cages were enriched with Shepard shacks (Shepard Specialty Papers), clear plastic tunnels (Braintree Scientific), swings (Datesand Group), and two different kinds of nesting material (Nestlet, Enviro‐dri; both Shepard Specialty Papers). Food and water were available ad libitum, and the light/dark cycle was 12/12 h with lights on at 7 am and off at 7 pm.

A total of 6 mice were used in two separate experiments (*Experiment 1*: *n* = 4; *Experiment 2*: *n* = 2). In *Experiment 1*, 4 mice underwent s.c. implantation surgery, after which oxygen measurements were performed every week. Mice were euthanized 2 weeks (*n* = 2) or 4 weeks (*n* = 2) after surgery by carbon dioxide overdose and cervical dislocation. Skin samples, including remnants of *Oxygel* implants, were dissected and fixed in 4% paraformaldehyde (Electron Microscopy Sciences) for 6 h before sucrose treatment (30%) and cryo‐embedding. In *Experiment 2*, 2 mice underwent implantation surgery, after which oxygen measurements were performed weekly starting 4 weeks after the surgery until euthanasia at 8 weeks. Visual post‐mortem examination of the s.c. area was performed to evaluate the presence of *Oxygel* residues (Figure S4).

For anesthesia induction, mice were transferred into a Plexiglas box filled with 2.0% isoflurane (Dechra Veterinary Products) in pure oxygen (Vet Flo Isoflurane Vaporizer, Kent Scientific). During preparation and surgery, mice were kept on a heating pad (37°C) to prevent hypothermia, and the anesthesia was maintained as 1.5% isoflurane/O_2_ using a nose cone. Preparation included application of eye ointment, subcutaneous (s.c.) injection of Ethiqa XR (1 mg/kg; buprenorphine extended‐release injectable suspension; Fidelis Animal Health) and shaving and disinfection (ethanol and povidone–iodine, Braunoderm, B. Braun) of the neck area.

At the U. Wisconsin site, nude male mice 6–8 weeks of age (Envigo, IN, USA) were used. Mice were housed in a pathogen‐free facility with controlled temperature and humidity under a 12‐h light/dark cycle, with ad libitum access to food and water. Animals were monitored daily for signs of distress, pain, or adverse events throughout the experimental period. All surgical and imaging procedures were performed under inhaled isoflurane anesthesia to minimize discomfort. For longitudinal imaging sessions, body temperature was maintained using a heated stage, and ophthalmic lubricant was applied to prevent corneal drying. At study completion, animals were euthanized using CO_2_ inhalation followed by cervical dislocation, consistent with the recommendations of the American Veterinary Medical Association (AVMA) Guidelines for the Euthanasia of Animals. No unexpected animal deaths occurred during the study, and all efforts were made to minimize animal suffering and reduce the number of animals used.

## 3 Results and Discussion

### 3.1 *Oxygels* Design

PEGs are generally considered nontoxic, biocompatible, biodegradable, and/or excretable polymers [44, 45]. In previous studies, metalloporphyrins with several functional groups were used as both functional dopants and cross‐linkers to form phosphorescent PEG‐based hydrogels [30, 31]. Although straightforward, this approach has two shortcomings. First, to form a fully cross‐linked matrix, the total number of functional groups on the cross‐linker molecules (*N*) in the mixture should match the number of the end groups on the bifunctional PEGs. Hence, the molar fraction of the cross‐linker (*n*) is *n* = 2/*N*, which for small numbers *N* translates into extremely high local concentrations of phosphorescent centers in the hydrogel matrix even after its complete swelling. At such high concentrations, triplet states are strongly quenched via processes involving nearby ground‐state porphyrin molecules, causing loss of emissivity, which, based on the reported photophysical properties, has been seen in some Pd tetracarboxyphenyl‐porphyrin‐based PEG hydrogels [30].

Second, a hydrated PEG shell is not capable of slowing down oxygen diffusion to a significant extent compared to water [5, 46, 47]. As a result, photoexcited triplets in the hydrated PEG hydrogel are quenched by diffusing oxygen molecules extremely rapidly, such that the phosphorescence can be detected only at very low oxygen concentrations. For broader applicability, impeding oxygen diffusion near phosphorescent centers would be desirable.

The latter issue can be resolved by including phosphorescent emitters into hydrophobic dendrimers, an approach that has been used in the construction of soluble oxygen probes, Oxyphors [5, 12, 13, 48]. In Oxyphors, phosphorescent Pt or Pd porphyrins are encapsulated in arylglycine (AG) dendrimers, whose periphery is modified with multiple hydrophilic PEG residues. By changing the dendrimer size, Stern–Volmer oxygen quenching constants of the probes can be tuned to achieve optimal sensitivity in the desired oxygen range [12, 49]. We reasoned that an Oxyphor without the external PEG layer could be covalently affixed within a cross‐linked PEG matrix, where its sensitivity would be tunable by varying the dendrimer generation/size. It should be mentioned that previously Pt porphyrins with dendritic arms have been incorporated in PEG‐based hydrogels [32, 33]; however, polyglutamic dendrimers employed in these studies are not capable of efficient shielding of porphyrins from oxygen, as has been shown previously [46, 49].

Arylglycine dendrimers are terminated by multiple carboxylic acid groups. A high number of peripheral functionalities (*N*) helps to reduce the molar fraction of the probe (*n*) in the gel, for example, *n* = 1/16 for a PEG‐based gel cross‐linked by Gen 2 polyarylglycine porphyrin‐dendrimers (*M* = 32) [12]. However, the ability to further dilute the probe is still desirable for complete prevention of self‐quenching and achieving maximal brightness. For that purpose, in the present design we used an optically neutral dendrimer as the main cross‐linker, while the porphyrin‐dendrimers were added as optically‐active dopants. By changing the ratio between the neutral cross‐linker and phosphorescent dopant, the properties of the gel could be tuned to maximize performance.

### 3.2 *Oxygels* Synthesis and Composition

**SCHEME 1:**
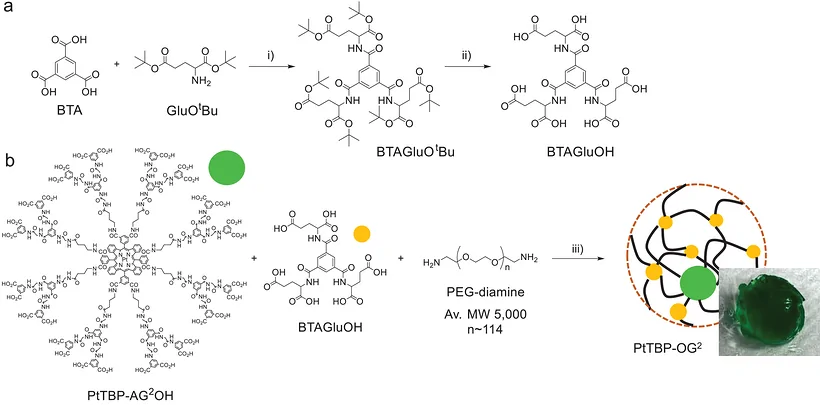
Syntheses of the optically neutral cross‐linker BTAGluOH (a) and *Oxygel* PtTBP‐OG^2^ (b). ^a^ Reagents and conditions: (i) HBTU (3.2 equiv), DIPEA (6.4 equiv), DMF, r.t., 24 h; (ii) TFA, DCM, (2:1) r.t., 3 h. Combined yield 86%. (iii) HBTU (2 equiv), DIPEA (2 equiv), DMF, rt.

The syntheses of the neutral cross‐linker and of *Oxygels* are illustrated in Scheme 1. The *Oxygels* consist of the backbone made of polyetheleneglycole diamine, H_2_N‐(CH_2_O)_n_CH_2_‐NH_2_ (Av MW 5,000; n∼114), cross‐linked by a dendritic optically‐neutral cross‐linker (BTAGluOH) and Pt tetrabenzoporphyrin‐polyarylglycine dendrimers PtTBP‐AG*^m^* OH (*m* = 0–2). The *Oxygels* (OG) are abbreviated by analogy with the parent dendrimers, PtTBP‐OG*^m^*, where *m* designates the generation of the arylglycine dendrimer [12].

The cross‐linker, BTAGluOH, was synthesized in two steps from benzene‐1,3,5‐tricarboxylic acid (BTA) (Scheme 1a) in 86% overall yield. Bulk quantities of BTAGluOH could be readily generated and stored for future use. The synthesis of metallotetrabenzoporphyrin‐arylglycine dendrimers was described in detail previously [12]. In the present work, along with the parent Pt octacarboxy‐tetraaryltetrabenzoporphyin (PtTBP), referred to as Gen 0 dendrimer, we used arylglycine (AG) dendrimers PtTBP‐AG*^m^* OH (*m* = 1, 2), which are terminated by 16 and 32 carboxylic acid groups, respectively. The hydrogels were synthesized via the peptide coupling reaction (Scheme 1b), rendering phosphorescent probes firmly affixed in the PEG matrix by multiple amide bonds.

Preliminary experiments revealed that the molar fraction of the phosphorescent probe in the gel should not exceed ∼0.01–0.02 due to the very high molar extinction coefficient of the PtTBP chromophore in the visible range (ε∼10^5^M^−1^cm^−1^ at 620 nm). At higher fractions (>0.02), the *Oxygels* became nearly opaque, precluding efficient excitation throughout the bulk of even small gel pieces (e.g., 1–2 mm across). Thanks to the optically neutral cross‐linker, BTAGluOH, the content of the probe in the gel could be readily adjusted without jeopardizing the gel's structural integrity.

To achieve complete cross‐linking, *Oxygels* were first formulated such that the total number of the carboxylic acid groups in the mixtures matched the number of the complementary amino groups in PEG‐diamines, while keeping the molar fraction of the probe constant. This strategy proved successful for PtTBP‐OG^0^, judged by the gel's appearance and its mechanical integrity. PtTBP‐OG^0^ could be easily cut to desired shape, did not fracture under pressure, and upon release of the pressure resumed the original shape. However, as the number of the carboxylic acid groups in PtTBP‐AG*^m^* OH increased (*m* = 1, 2), and, consequently, the fraction of the neutral cross‐linker had to be lowered to keep the numbers of carboxyls and amino groups equal, the gels became mechanically less stable (soft, easily breakable), particularly PtTBP‐OG^2^, pointing towards incomplete cross‐linking of the PEG network. On the other hand, when the fraction of the neutral cross‐linker was increased, thereby reducing the content of the probe, the gel again became firm and robust (Tables 1 and S1, entries 1–3).

**TABLE 1:** Properties of *Oxygels* PtTBP‐OG*^m^* (*m* = 0–2) used in structural and photophysical studies ^a^.

#	*m*	*N* ^b^	*N*_CO2H_/*N*_NH2_ ^c^	*SR*, ^d^	*f*_p_,% ^e^	*f*_H2O_,% ^f^	*τ*_0_, µs ^g^	*τ*_air_, µs^h^	*k*_q_mmHg^−1^s^−1^ ^i^	*k*_q_ (soluble)mmHg^−1^s^−1^ ^j^
1	0	8	1.015	54 ± 2	0.36	98.4	37.7 ± 0.5	5.5	1147.5 ± 0.8	1208.2
2	1	16	1.07	38 ± 1	0.99	97.9	39.1 ± 0.7	8.1	776.0 ± 0.7	521.3
3	2	32	1.18	23 ± 2	1.90	95.3	45.1 ± 0.4	16.6	217 ± 0.4	171.5

^a^ All *Oxygels* were synthesized using PEG‐diamine (100 mol. eq), BTAGluOH (32 mol. eq.), and PtTBP‐AG*^m^* OH (1.375 mol. eq).

^b^ Number of carboxylic acid groups in PtTBP‐AG*^m^* OH dendrimers.

^c^ Ratio between carboxylic acid and amino groups.

^d^ Swelling ratio in water at equilibrium. The s.d. in the determination of *SR* was estimated based on three independent measurements.

^e^ Fraction of PtTBP‐AG*^m^* OH dendrimer (by weight) in dry *Oxygels*.

^f^ Fraction of water (by weight) in swollen *Oxygels*.

^g^ Phosphorescence lifetimes at zero oxygen in aqueous phosphate buffer (20 mM, pH 7.2, 23°C).

^h^ Phosphorescence lifetimes at air saturation in aqueous phosphate buffer (20 mM, pH 7.2, 23°C).

^i^ Oxygen quenching constants (see Supporting Information for details).

^j^ Oxygen quenching constants for soluble Oxyphors PtP‐AG*^m^* PEG (*m* = 0‐2) (from ref. [12]).

The observed behavior is consistent with the peripheral crowding effect common for large dendrimers [50], causing some of their peripheral groups to be less accessible and, therefore, less reactive. In contrast, fewer peripheral carboxyls in the neutral cross‐linker BTAGluOH (only 6) facilitated more complete reaction with PEG‐amines. Reducing the molar fraction of the optically active component (probe) below 0.01 was not desirable from the application's perspective. Thus, in the *Oxygel* formulations used in the optical experiments (Table 1) the molar fractions of the probe and of the neutral cross‐linker were fixed (∼0.01 and ∼0.24, respectively), so that in PtTBP‐OG^1^ and PtTBP‐OG^2^ the ratio between the carboxyl and amino group (*N*_CO2H_/*N*_NH2_) exceeded unity, resulting in *Oxygels* with high mechanical stability and, simultaneously, high concentration of the optical dopant to ensure strong phosphorescence.

### 3.3 Structural and Photophysical Properties of *Oxygels*

The compositions of the studied *Oxygels* as well as their photophysical and oxygen quenching properties are summarized in Table 1 (for additional formulations see Table S1).

**FIGURE 1:**
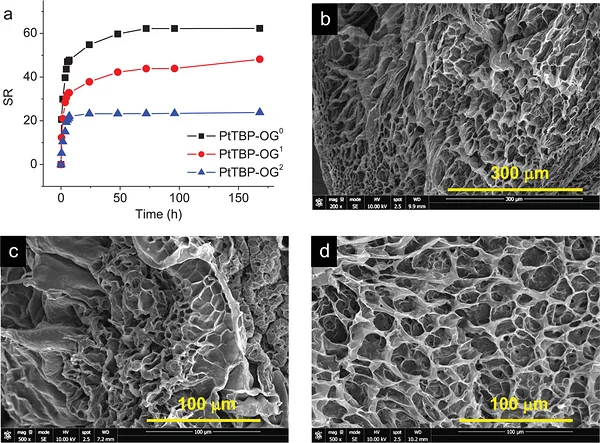
(a) Swelling of *Oxygels* PtTBP‐OG^0^, PtTBP‐OG^1^, and PtTBP‐OG^2^ in aqueous buffer quantified by weight‐based *swelling ratio (SR)* (see Supporting Information for details). Scanning electron microscopy (SEM) images of PtTBP‐OG^0^ (b), PtTBP‐OG^1^ (c), and PtTBP‐OG^2^ (d) after swelling and freeze‐drying.

Swelling behavior is an important indicator of hydrogels' structural integrity, commonly used to evaluate suitability of long‐term implants [51]. Swelling of *Oxygels* was quantified using a weight‐based *swelling ratio* (*SR*), defined as the relative increase in the weight of the gel upon absorption of water (see Supporting Information 3). A piece of dry *Oxygel* was placed in an aqueous medium, and the weight of the sample was measured at selected time intervals (Figure 1a). Since linear dimensions of a gel are proportional to the cubic root of its volume, which is proportional to its weight, the SR reflects changes in the gel size as well. The measurements revealed that PtTBP‐OG^2^ reached saturation after ∼30–60 min, after which its mass remained constant until the end of the experiment. *Oxygels* PtTBP‐OG^0^ and PtTBP‐OG^1^ took longer to swell, but once their weights reached the respective plateaus, they remained constant as well. The observed stability suggests that *Oxygels* are likely to maintain their size and structural integrity after implantation into tissue, which is important for the target sensing application.

The equilibrium SR at saturation shows a marked dependence on the dendrimer generation, decreasing from 54 (*m* = 0) to 38 (*m* = 1), to 23 (*m* = 2). Potentially, gels’ hydrophobicity could affect swelling behavior, since hydrophobic matter would be expected to repel water from the gel interior. In this regard, the molecular weights of PtTBP‐AG*^m^* dendrimers (probes), which are the most hydrophobic components of the *Oxygels*, increased in the series, and so did their fractions by weight (*f*_p_; Table 1), although the latter increase was quite small, only from 0.36% to 1.9%. Simultaneously, the ratio between the amino and carboxylic acid residues (*N*_CO2H_/*N*_NH2_) also increased from 1.01 to 1.18, which could have its own effect on the swelling behavior. Additional experiments (Supporting Information 3; Table S1 and Figure S1) performed with PtTBP‐OG^2^ revealed that decreasing the hydrophobic fraction *f*_p_ from 0.84% to 0.44% while keeping the ratio *N*_CO2H_/*N*_NH2_ constant (equal to 1.0) did cause an increase in the SR, but only by ∼20%. On the other hand, increasing both *N*_CO2H_/*N*_NH2_ (from 1.0 to 1.18) and *f*_p_ (from 0.84 to 1.9) had virtually no effect on the *SR* value, which remained ∼20. This contrasts with a ∼2.5‐fold increase in *SR* seen in the dendrimer series (Table 1) for the same increase in *N*_CO2H_/*N*_NH2_ and *f*_p_. Clearly, more studies are needed to understand the role of hydrophobic dopants in *Oxygel*’s swelling behavior.

The morphologies of the fully hydrated and subsequently freeze‐dried hydrogel networks were examined using scanning electron microscopy (SEM; Figure 1b–d). SEM images revealed uniform, porous, sponge‐like structures with the average pore sizes declining with an increase in the generation number, that is, from ∼50 µm for PtTBP‐OG^0^ to 30 µm for PtTBP‐OG^1^ and to 20 µm for PtTBP‐OG^2^. This change correlates with the increase in the *SR* (Figure 1a), suggesting that the networks with larger pores are able to absorb larger amounts of water. Notably, in all cases the *SR* was large enough to ensure high optical transparency of the *Oxygels*, which is expected for materials that are composed essentially of liquid water. Indeed, the water content (by weight) in the hydrated *Oxygels* (*f*_H2O_, Table 1) varied in the range of 96.0%–98.4%. Another important advantage of the highly porous architecture is that it facilitates diffusion of analytes into the gel, including oxygen, thus aiding equilibration between oxygen outside and within the gel and ensuring fast response of the sensors to oxygen changes in the environment.

As expected, the optical absorption and emission spectra of the synthesized *Oxygels* are nearly identical to those of the PtTBP‐dendrimers [12]. In the visible range, all *Oxygels* exhibit characteristic sharp B and Q bands of the parent PtTBP chromophore (e.g., *λ*_B_ = 432 nm and *λ*_Q_ = 621 nm for PtTBP‐OG^2^) and phosphoresce in the near‐infrared part of the spectrum (*λ*_max_∼780 nm) (Figure S2). Compared to PdTBP complexes employed in some hydrogels previously [40], PtTBP‐based oxygen probes offer much higher brightness [12], which is critical for deep‐tissue sensing.

**FIGURE 2:**
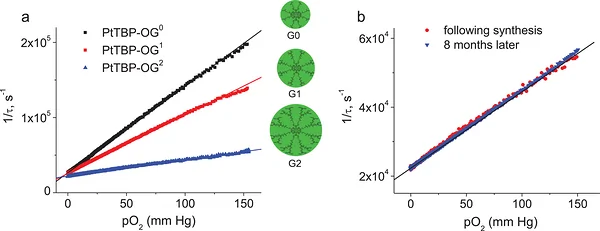
Oxygen quenching properties of *Oxygels*. (a) Stern–Volmer phosphorescence oxygen quenching plots of *Oxygels* containing PtTBP‐AG^m^ dendrimers (*m* = 0–2) measured in phosphate buffer (50 mM, pH 7.4, 23°C). *τ* denotes the phosphorescence decay time. The slopes of the linear fits (solid lines) correspond to the oxygen quenching constants *k*_q_ (Table 1). (b) Stern–Volmer oxygen quenching plots of PtTBP‐AG^m^ measured immediately after the synthesis and 8 months later after storage in hydrated state (phosphate buffer, 50 mM, pH 7.4, 4°C).

The effect of the dendritic shell surrounding the phosphorescent center (PtTBP) is illustrated in Figure 2a. Quenching of porphyrin triplet states by oxygen can be formally classified as a triplet‐triplet annihilation process, and it occurs essentially in the diffusion‐controlled regime. According to the Stern–Volmer model [52], the dependence of the phosphorescence lifetime (*τ*) on the concentration of oxygen (or pO_2_) is given by the linear form, 1/*τ* = 1/*τ*_0_ + *k*_q_×pO_2_, where *k*_q_ is the bimolecular quenching rate constant, and *τ*_0_ is the lifetime in the absence of oxygen. In Figure 2a, the slopes of the Stern–Volmer plots, which represent constants *k*_q_, reflect the rates of oxygen diffusion in the vicinity of the triplet chromophore. In PtTBP‐OG^0^, the porphyrin has no dendritic shell, and hence the slope of the plot corresponds to oxygen diffusion in the bulk of the hydrated PEG matrix. Folded dendritic branches in PtTBP‐OG^1^ and PtTBP‐OG^2^ impose diffusion barriers for oxygen, and constants *k*_q_ decrease accordingly, that is, by threefold for PtTBP‐OG^2^ compared to PtTBP‐OG^0^.

It is interesting to compare the quenching constants *k*_q_ obtained for the *Oxygels* with those of similar soluble probes, in which porphyrins are surrounded by just a single PEG layer, as opposed to the cross‐linked macroscopic PEG network. A complete dataset was available for a series of dendrimers having a non‐extended Pt porphyrin as a core, PtP‐AG*^m^* PEG (*m* = 0–2) (Supporting Information 5) [12]. While the absolute *k*_q_ values may be affected by the nature of the phosphorescent center (even for a diffusion‐controlled quenching reaction), relative changes introduced by dendrimers should be comparable between the PtP‐ and PtTBP‐derived series. Remarkably, the decrease in the quenching rate with the dendritic generation in the PtP‐AG*^m^* PEG series (Table 1, last column) is similar or even more pronounced than that among the *Oxygels* (PtTBP‐OG*^m^*), indicating that PEG shell, whether it is just a single layer or the entire macroscopic hydrogel network, has virtually no effect on the efficiency of oxygen quenching. This is consistent with quenching being limited by the rate of oxygen diffusion through the folded hydrophobic AG dendrimer as well as with the large size of the pores in the hydrated *Oxygel* network (Figure 1b–d), where oxygen diffusion occurs essentially at the same rate as in water.

A probe with the highest quenching constant would have the largest range of phosphorescence lifetimes over a chosen range of oxygen concentrations, and hence, in theory, it should have the highest sensitivity for oxygen. However, because phosphorescence is progressively quenched as oxygen concentration increases, probes with too high constants *k*_q_ can produce measurable signals only under the state of hypoxia, while being inadequate for measurements at normoxic levels. Indeed, due to the inverse relationship between the decay time and pO_2_, the whole range of near‐normoxic oxygen concentrations would become packed into a narrow range of lifetimes for probes with high *k*_q_’s. At the same time, accurately measuring these lifetimes would be difficult because most of the signal would be quenched by oxygen. Thus, appropriately attenuating quenching constants would be beneficial for broader applications, as discussed earlier [5, 12, 13, 48]. For the CELI tests (see below), we used *Oxygel* PtTBP‐OG^2^, which exhibits lifetimes of 16.6 µs at air saturation (*τ*_air_) and 45.1 µs in the absence of oxygen (*τ*_0_) at 23°C (Table 1). At physiological temperature (37°C), *k*_q_ increases by ∼50% (Supporting Information 6 and Figure S3a). Nevertheless, PtTBP‐OG^2^ is still capable of producing robust signals at all physiologically relevant oxygen levels. *Oxygels* of earlier generations (*m* = 0, 1) are less practical at higher (normoxic) pO_2_’s due to their considerably higher quenching constants *k*_q_.

From the application's perspective, stability of a sensor over time is an important parameter, especially for longitudinal imaging. In this regard, calibration constants of PtTBP‐OG^2^ were found to be practically unchanged after storing the gel for >8 at 4°C relative to the measurements performed immediately after the synthesis (Figure 2b), demonstrating high stability and integrity of the gel. Likewise, the Stern–Volmer plots were nearly identical after ∼3 days of storage at 37°C. When dried by lyophilization and then rehydrated, oxygen quenching parameters were found to be practically unchanged as well (Figure S3a).

### 3.4 *Oxygels* Biocompatibility and Biodegradation

Evaluation of *Oxygel* degradation and histological analyses were performed in mice upon subcutaneous implantation of PtTBP‐OG^2^ (pellets ∼1–2 mm diameter) at weeks 2, 4, and 8 post‐implantation (Supporting Information 7). Prior to excision of the implanted *Oxygels*, phosphorescence signals were measured via surface excitation/detection using a fiber‐optic phosphorometer, as described earlier [12, 13]. At week 2, the hydrogel was firmly attached to the subcutis and retained its original dimensions. By week 4, the hydrogel was still attached, but its swelling was apparent (Figure S4), which could be caused by partial hydrolysis of the internal amide bonds and concomitant decline in the degree of cross‐linking. Indeed, examination at week 8 revealed no identifiable traces of the implant; however, phosphorescence from the area where the implant was located was still detectable, suggesting that PtTBP‐AG^2^ dendrimer remained intact in spite of the hydrolysis of the surrounding PEG matrix. Up to week 4, the signals emitted from the implant locations were strong, and the pO_2_ readings were within the physiological range (Figure S5).

**FIGURE 3:**
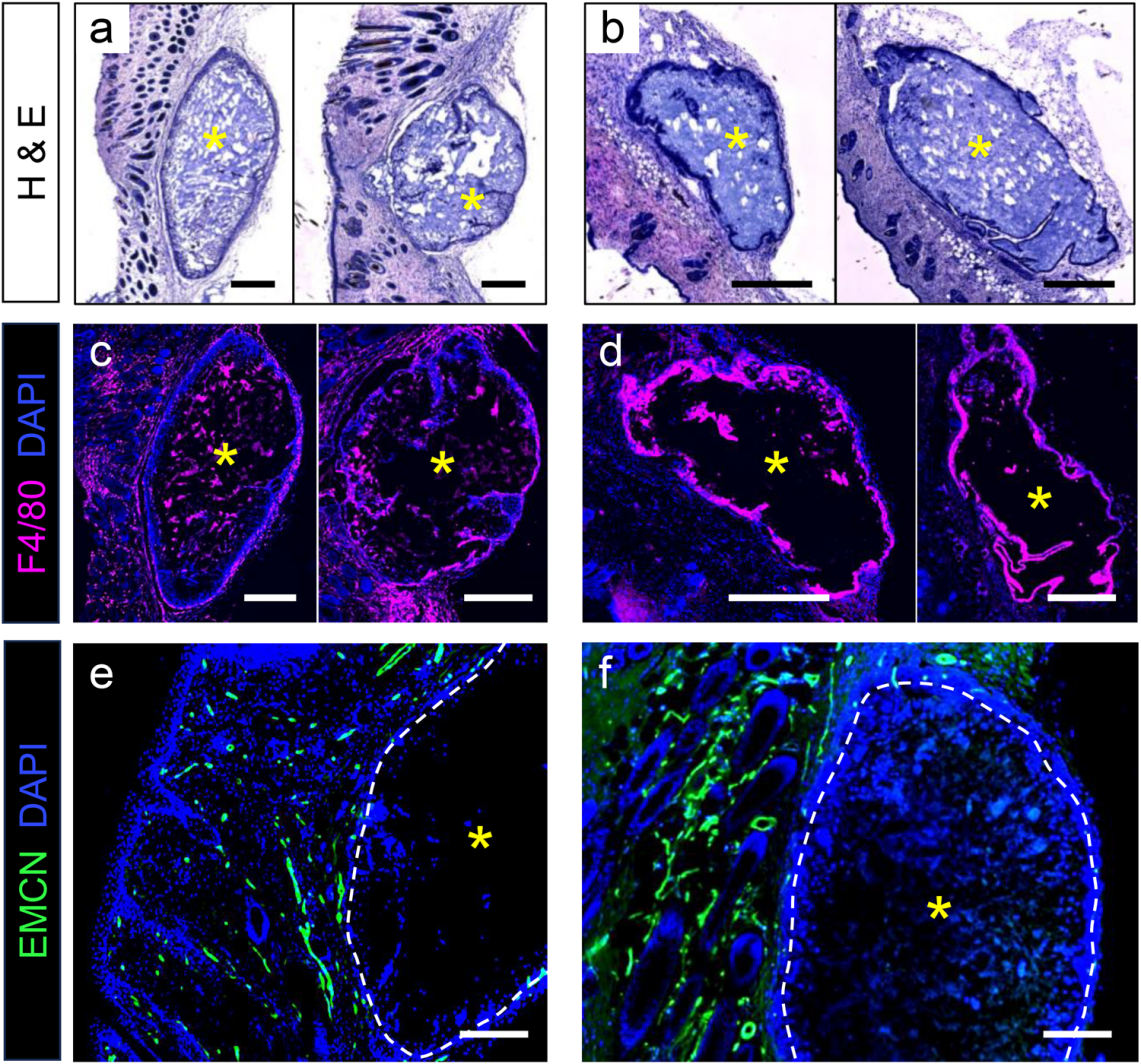
Tissue response to implantation of *Oxygels*. H&E staining 2 (a) and 4 weeks (b) post‐implantation. F4/80 staining for the presence of macrophages 2 (c) and 4 weeks (d) post‐implantation. EMCN staining for angiogenesis 2 (e) and 4 weeks (f) post‐implantation. Areas corresponding to *Oxygel* pieces are marked with asterisks and circled by dashed lines in (e) and (g). Scale bars: (a–d) 500 µm, (e,f) 200 µm.

Biocompatibility of *Oxygel* PtTBP‐OG^2^ was assessed via longitudinal measurements of the tissue foreign body response. First, standard hematoxylin and eosin (H&E) staining, which causes nuclei to appear blue and cytoplasmatic proteins and extracellular fibers pink, was used to examine tissue morphology near the implantation site. Thin capsule‐like cell layers formed around the implanted *Oxygel* pellets after 2 weeks (Figure 3a) and became more pronounced and thickened after 4 weeks (Figure 3b). However, there was no indication of elevated infiltration of inflammatory cells into the surrounding tissue, and neither could we detect any foreign body giant cells (FBGCs), whose presence is typically associated with inflammatory responses caused by foreign objects, suggesting that the *Oxygel* induced only a mild immune reaction.

Second, the excised tissue was examined for the presence of macrophages that are typically activated during the response to foreign biomaterials. Immunostaining was used to detect a specific glycoprotein, F4/80, expressed on the surfaces of murine macrophages. Two weeks post‐implantation, F4/80‐positive cells were found to be heterogeneously distributed at the periphery of the implanted hydrogels in the surrounding cell layers (Figure 3c; pink stain). At week 4, a pronounced layer of F4/80‐positive cells formed around the *Oxygel* implant (Figure 3d), reflecting active interaction at the material interface and suggesting a moderate foreign body response. Such reactions are well documented for PEG‐based hydrogels, indicating that inclusion of Oxyphor molecules and dendritic cross‐linker (BTAGluOH) into the gel did not exacerbate the response; rather, it was moderate compared to what has been reported in the literature for other PEG‐based hydrogels [53, 54].

Lastly, endomucin (EMCN) staining was performed to determine whether implantation induced neo‐vascularization. Immunofluorescence staining for EMCN, a glycoprotein present at the luminal side of the capillary endothelium, is used to detect angiogenesis. No pronounced infiltration of vessels into the gel could be detected either at weeks 2 or 4 post‐implantation (Figure 3e,f). There appeared to be more vessels in the periphery at week 4, which could indicate matrix remodeling by the F4/80‐positive macrophages. This observation is consistent with previous reports indicating that PEG‐based materials generally require additional modification to support vascular infiltration [55].

In parallel with staining for F4/80 and EMCN, DAPI was used to nonspecifically stain the cells' nuclei (Figure 3c–f), revealing higher cellular density near the implantation site, consistent with experiments shown in Figure 3a–d.

Taken together, our results show that implantation of *Oxygels* induces a moderate initial foreign body immune response, while the phosphorescence signals remain stable over at least 4 weeks period, during which *Oxygels* could be reliably used for local tissue oxygen sensing. In the future, complete bio‐degradation pathways of *Oxygels* will need to be studied, should these materials be considered for clinical translation. It is likely that remnants of the PEG matrix, which constitutes the bulk of *Oxygels*, would be excreted via kidney dialysis. PtTBP‐dendrimers could be excretable as well, although the concentration of these fragments in relation to the total body weight would be exceedingly low (picomolar range), making it unlikely that they would cause any toxicity even if retained indefinitely.

### 3.5 In Vivo Cherenkov‐Excited Luminescence Imaging

**FIGURE 4:**
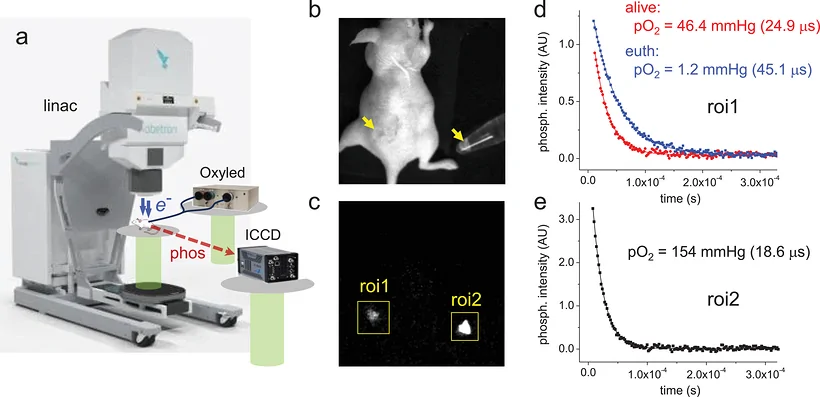
CELI of oxygen with subcutaneously implanted *Oxygel* PtTBP‐OG^2^. (a) Setup for CELI with two data acquisition systems: ICCD camera and single‐detector fiberoptic phosphorometer. (b) Mouse with subcutaneously implanted *Oxygel* piece (20 mg) and an Eppendorf tube with *Oxygel* piece. The locations of the *Oxygel* pieces are marked with yellow arrows. (c) Wide‐field phosphorescence intensity image obtained using the ICCD camera upon Cherenkov excitation by an electron pulse (0.25 Gy, acquisition delay 5 µs). Regions of interest (roi's) corresponding to the implanted and reference *Oxygels* (as shown in b) are shown in yellow squares. (d) Phosphorescence decays from the implanted *Oxygel* (roi1) recorded using the fiberoptic phosphorometer. The phosphorescence decay times represent tissue pO_2_ in an alive (46.4 mmHg) and later euthanized (1.2 mmHg) mouse. (e) Phosphorescence decay from the reference *Oxygel* (roi2) equilibrated with air.

The setup for CELI (Figure 4a and Supporting Information 8) used in our experiments has been described previously [20]. The radiation source was an electron linear accelerator (linac) operating in FLASH mode. In the present study, phosphorescence emitted by the object (a mouse with implanted *Oxygel*) was detected using two methods. First, to perform spatially resolved CELI, we used an intensified charge‐coupled device (ICCD) camera, whose gate was synchronized with linac pulses, such that the integration on the CCD began after a pre‐selected delay relative to the pulse, thereby separating phosphorescence from the bright Cherenkov flash. Second, for more accurate phosphorescence lifetime information for oxygen quantification, we used a single‐detector phosphorometer (Oxyled) [12, 13, 56]. The detector was coupled to the object by a single‐mode fiber (4 mm in diameter), and its signal acquisition procedure was synchronized with linac pulses as well, whereby phosphorescence decays were collected upon excitation by a single pulse (7.9 Gy/pulse). In addition, using a light‐emitting diode, phosphorescence could be excited by conventional light pulses from the phosphorometer.

In a test experiment, a piece of PtTBP‐OG^2^ (∼20 mg) was implanted subcutaneously in a mouse, and a small Eppendorf tube containing a piece of *Oxygel* of approximately the same size was placed next to the animal to provide a control signal (Figure 4b). In the integrated intensity image acquired using a 5 µs delay after the electron pulse, both phosphorescence sources are clearly detectable (Figure 4c; regions of interest roi1 and roi2), while the signal from roi2 is expectedly brighter, since the *Oxygel* in the tube was not covered by the skin. Remarkably, the phosphorescence signals obtained upon excitation by single electron pulses (7.9 Gy) were characterized by rather high SNR sufficient for resolving pO_2_ with ±2.5 mmHg accuracy (in the mean tissue pO_2_ region, near 45 mmHg) (Figure 4d). In the future, light collection efficiency can be significantly improved (e.g., by increasing the fiber diameter and/or using several fibers pointing at the tissue area and having a common end coupled to the detector), further increasing the accuracy in pO_2_ determination, and/or allowing for measurements at higher depths. Based on the comparisons with the earlier reported transillumination [13] and tomographic [15] phosphorescence lifetime imaging experiments, we speculate that single‐electron‐pulse CELI measurements with *Oxygels* should be capable of oxygen sampling at depths of >1 cm. The relevant experiments are underway and will be reported separately.

After the mouse was euthanized, tissue pO_2_ sharply decreased, as expected, to essentially anoxic levels as a consequence of halted blood flow. This was reflected by a longer lifetime of the phosphorescence decay (Figure 4d). At the same time, the lifetime of the reference decay, corresponding to the *Oxygel* in the tube (Figure 4e; roi2), remained constant, reflecting oxygenation in the surrounding solution (PBS) equilibrated with air. All decay lifetimes were reproduced by using conventional surface light excitation/APD detection, allowing for cross‐validation of the performed excitation/signal retrieval methods.

**FIGURE 5:**
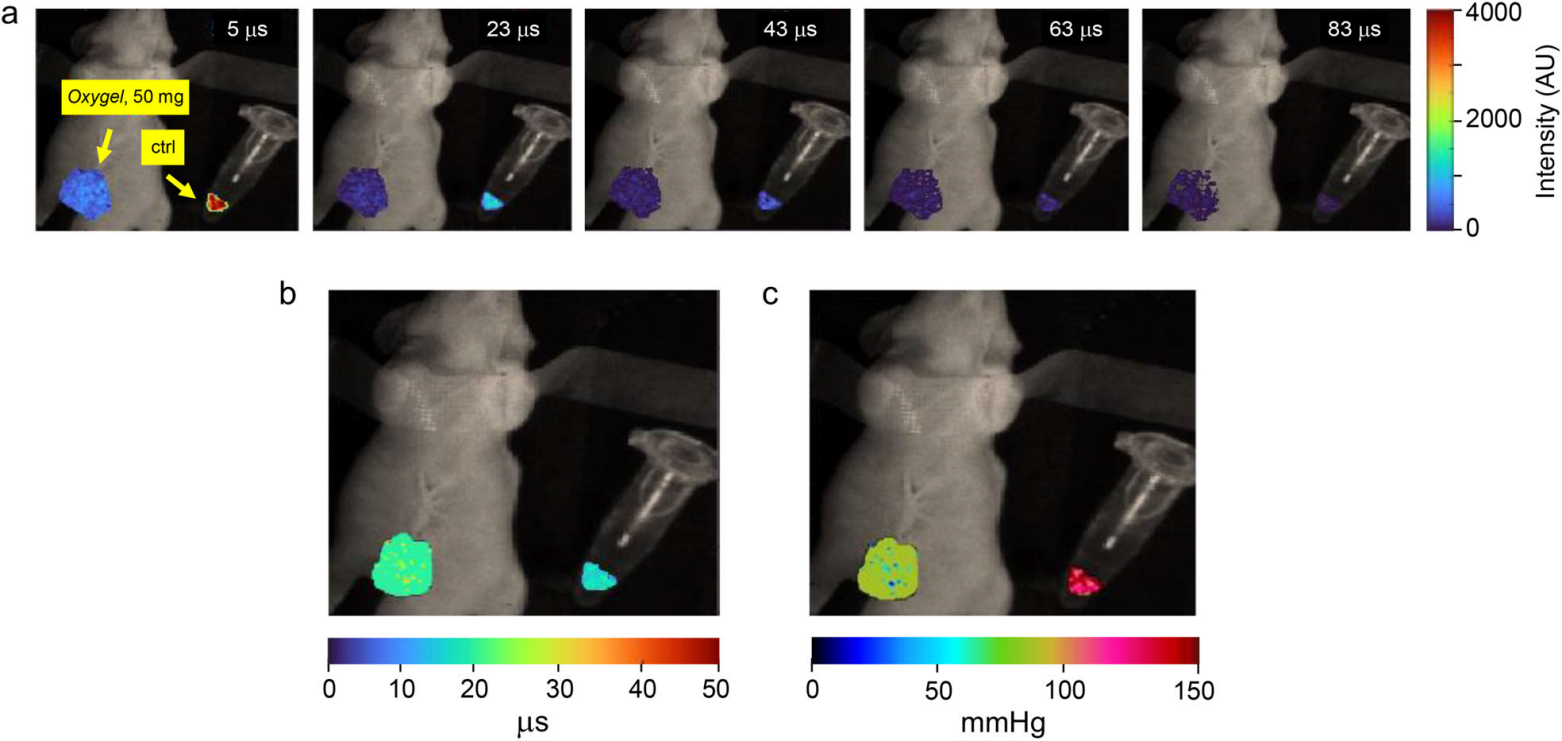
CELI of oxygen with *Oxygel* PtTBP‐OG^2^ (50 mg) implanted abdominally under the intestines ∼3 mm under the surface. An Eppendorf tube with *Oxygel* piece was positioned next to the animal. (a) Phosphorescence intensity images integrated on the CCD after selected delays. In total, 40 images were collected with delays incrementing in 2 µs steps starting with 5 µs. Excitation was by radiation pulses, 0.25 Gy/pulse; total dose 10 Gy. (b) Phosphorescence lifetime image obtained by pixel‐wise fitting of the intensity images by single‐exponentials. (c) Image of the partial pressures of oxygen (pO_2_) derived from the phosphorescence lifetime images using Stern–Volmer calibration constants for PtTBP‐OG^2^ measured at 37°C.

Camera gating could also be used for generating sequences of images with different delay times for subsequent pixel‐wise fitting to calculate phosphorescence lifetime images, which in turn could be converted into images of partial pressures of oxygen (pO_2_ image). This type of phosphorescence lifetime imaging has been used broadly in the past with soluble *Oxyphors* (see, e.g., refs. [13, 15]), including CELI of oxygen [24, 57]. Although in the present implementation, the camera was used primarily to visualize the locations of the *Oxygel* implants and obtain information about their integrity, it also allowed us to quantify pO_2_ in the tissue surrounding *Oxygels*, even when they were located deeper under the surface. In an example experiment, a piece of *Oxygel* was implanted in the mouse abdomen under the intestines, ∼3 mm under the surface (Figure 5). In this case, the implant was chosen to be larger (50 mg), considering its deeper location. Phosphorescence was excited by linac pulses (0.25 Gy/pulse), and after each pulse, the emitted signal was integrated on the CCD with delays incremented in 2 µs steps (Figure 5a). In total, 40 images were collected, resulting in a total dose of 10 Gy. The computed lifetime image (Figure 5a) and pO_2_ image (Figure 5c) qualitatively represent oxygenation in the respective environments, although the pO_2_ values corresponding to the implant appear to be somewhat higher than expected for the abdominal cavity (20‐30 mmHg).

Based on our current results, pO_2_ quantification using a single‐detector instrument (Figure 4) offers higher accuracy, which is primarily due to the higher efficiency of phosphorescence collection with a fiber positioned next to the animal than imaging it with a distant camera. Although using a single detector and a single *Oxygel* implant spatially resolved images of oxygen cannot be obtained, for clinical monitoring during RT, accurate single‐point measurements at depth, representing average oxygenation in the volume surrounding the sensor, could be highly informative, whereas such measurements are not currently available by any other method. In this regard, our present experiments mimic the scenario occurring in a new type of RT modality, termed FLASH RT [58, 59]. In FLASH RT, radiation is delivered at ultrahigh dose rates such that, in the case of electron FLASH, a clinical dose (e.g., 10 Gy) is contained within a single microsecond‐long pulse, as opposed to long (minutes) trains of low‐intensity pulses used for conventional delivery. FLASH RT is currently one of the most intensely discussed topics in radiation oncology due to its remarkable ability to cause less damage to healthy tissues, compared to conventional RT, while maintaining equal antitumor effectiveness. The mechanism of this so‐called FLASH effect, although not established, most likely involves molecular oxygen [60, 61]. Hence, the ability to assess tumor and/or surrounding tissue oxygenation during application of FLASH RT would be highly beneficial for both its clinical translation and elucidation of the underpinning mechanisms.

Overall, the performed CELI tests demonstrate the feasibility of deep tissue oxygen sampling using *Oxygels* as local phosphorescent sensors in combination with excitation by radiation pulses commonly used in clinical RT. These results are particularly valuable in view of rapidly developing FLASH RT, where information about tissue oxygenation at the time of delivery could help unravel the mystery of the FLASH effect and facilitate clinical translation.

## 4 Conclusions

In this contribution, we developed phosphorescent PEG‐based hydrogels with tunable oxygen quenching properties and validated their performance in tissue oxygen measurements by CELI. Our design builds upon the previous achievements in the field, but offers several new features, which proved to be highly useful for the target oxygen imaging application. First, phosphorescent chromophores were embedded within the hydrogel network not directly, but encapsulated in hydrophobic arylglycine dendrimers, and the latter were cross‐linked within the PEG matrix. Encapsulation into dendrimers enabled optimization of the *Oxygels*’ sensitivity to maximize performance in the physiological oxygen range. Second, in *Oxygels*, porphyrin‐dendrimers (probes) play the role of dopants, while the main cross‐linker is an optically neutral dendrimer. Changing the ratio between the cross‐linker and the probe makes it possible to independently vary the gel's rheological properties and adjust the concentration of the probe to achieve the best performance.

Biocompatibility studies showed that *Oxygels* do not induce prolonged immune reactions and/or inflammation but only a minor initial foreign body response. For at least 4 weeks post‐implantation, *Oxygels* maintained structural integrity and, despite gradual swelling, produced robust phosphorescence signals. Considering that the hydrated PEG matrix does not affect phosphorescence oxygen quenching constants, which are defined solely by the dendritic shells, the structural integrity of *Oxygels* confirms that they maintain their ability to report on local tissue oxygenation quantitatively, at least until the PEG matrix is fully biodegraded.

CELI tests showed that small *Oxygel* implants allow oxygen measurements in vivo at depth using Cherenkov light from electron pulses for phosphorescence excitation. The depth of sampling is ultimately a function of the phosphorescence collection efficiency and the signal strength, both of which can be improved as the technology develops further. For example, probes based on brighter porphyrins, such as PtTAPIP [62], could be used, while doping could be further optimized specifically for excitation by Cherenkov light. At the same time, using a multi‐fiber signal collection approach could substantially increase the amount of phosphorescence reaching the detector.

Perhaps, the most significant drawback of the *Oxygel* technology in its present form, common for other gel‐based tissue sensors [37], is the necessity to perform physical implantation of solid gel pieces, which is invasive and perturbs tissue oxygenation at the measurement site. This perturbation could be partially mitigated by delaying measurements relative to the implantation procedure, letting the blood vessels network damaged during the implantation recover. However, minimizing the intrusion is always highly desirable, as it would diminish risks of other potentially unwanted outcomes. In this regard, creating phosphorescent materials that could be injected into tissue as liquids using thin needles and form gels immediately at the injection site would be the next challenge for the *Oxygel* technology that would ultimately bring it closer to clinical translation.

## Author Contributions

**Simin Belali**: conceptualization, methodology, investigation, validation, and writing – original draft. **Marien Iliza Ochoa Mendoza**: methodology, visualization, formal analysis, and writing – reviewing & editing. **Matthew S. Reed**: methodology, visualization, formal analysis, and writing – reviewing & editing. **Danylo Mykhalchuk**: methodology and investigation. **Annemarie Lang**: methodology, investigation, visualization, formal analysis, validation, and writing – reviewing & editing. **Joel D. Boerckel**: supervision, resources, and writing – review & editing. **Brian W. Pogue**: conceptualization, methodology, writing – review & editing, supervision, resources, and funding acquisition. **Sergei A. Vinogradov**: conceptualization, methodology, formal analysis, writing – review & editing, supervision, project administration, resources, and funding acquisition.

## Conflicts of Interest

S.A.V. holds a patent for the Oxyphor probes technology related to this work. There are no other conflicts of interest to declare.

## Supporting information




**Supporting File**: adhm71586‐sup‐0001‐SuppMat.pdf.

## Data Availability

The data that support the findings of this study are available from the corresponding author upon request.
