# Supplementary material for: Oxygels: Tunable Phosphorescent Hydrogels for Cherenkov‐Excited Luminescence Imaging (CELI) of Oxygen
Source: Adv Healthc Mater. 2026 Aug 12;15(34):e71586. doi: 10.1002/adhm.71586 (PMC13568940; doi:10.1002/adhm.71586)

Oxygels: Tunable Phosphorescent Hydrogels for Cherenkov-Excited Luminescence Imaging (CELI) of Oxygen

Supporting Information

Simin Belali,[a,b] Marien Iliza Ochoa Mendoza,[c,d] Matthew S. Reed,[c,d] Danylo Mykhalchuk,[a,b] Annemarie Lang,[e,f] Joel D. Boerckel,[e,g] Brian W. Pogue,[c,d] Sergei A. Vinogradov[a,b]*

[a] *Department of Biochemistry and Biophysics, Perelman School of Medicine, University of Pennsylvania, Philadelphia, PA 19104, USA*

[b] *Department of Chemistry, School of Arts and Sciences, University of Pennsylvania, Philadelphia, PA 19104, USA*

[c] *Department of Medical Physics, School of Medicine, University of Wisconsin-Madison, Madison, WI 53705, USA*

[d] *Thayer School of Engineering, Dartmouth College, Hanover, NH 03755, USA*

[e] *Department of Orthopaedic Surgery, Perelman School of Medicine, University of Pennsylvania, Philadelphia, PA 19104, USA*

[f] *Department of Orthopaedic Surgery, University of Michigan Medical School, Ann Arbor, MI 48109, USA*

[g] *Department of Bioengineering, School of Engineering and Applied Science, University of Pennsylvania, Philadelphia, PA 19104, USA*

E-mail: vinograd.upenn@gmail.com

Keywords: oxygen, phosphorescence, hydrogel, porphyrin, Cherenkov-excited luminescence, Oxyphor.

Table of Contents

1. General information S3

2. Synthesis S4

3. *Oxygel* swelling S6

4. Optical absorption and emission spectra of *Oxygels* S7

5. Structures of Pt porphyrin-dendrimers PtP-AG^{m}PEG (m=0-2) S8

6. Oxygen quenching plots S9

7. *Oxygel* biocompatibility and biodegradability studies S12

8. Cherenkov-Excited Luminescence Imaging (CELI) S12

9. References S14

10. NMR spectra S15

1. General information

Pt octacarboxy-tetraphenyltetrabenzoporphyrin (PtTBP) was synthesized as described previously[1] and converted to PtTBP-AG^0OH (i.e. PtTBP with amended ethyl-4-aminobutyrate extension arms) using the same method as applied in the case of analogous PdTBP-AG^0OH.[2] PtTBP-AG^1OH and PtTBP-AG^2OH porphyrin-dendrimers were synthesized using PdTBP-AG^0OH and H_2N-AG^1(OBu) and H_2N-AG^2(OBu) dendrons as described previously.[1, 2]

All solvents and reagents were purchased from standard commercial sources (Sigma-Aldrich, Fisher Scientific) and used as received. Poly(ethylene glycol) diamine (NH_2-PEG-NH_2), Av. MW 5000, was obtained from Laysan Bio. 1,3,5-Benzenetricarboxylic acid, L-glutamic acid di-*t*-butyl ester hydrochloride, 2-(1H-benzotriazol-1-yl)-1,1,3,3-tetramethyluronium hexafluorophosphate (HBTU) and N,N-diisopropylethylamine (DIPEA) were obtained from Sigma-Aldrich. Thin-layer chromatography was performed using aluminum-backed silica gel TLC plates (SiliaPlate™ 200 µm, with F254 indicator; Silicycle). Column chromatography was performed on Silicycle Silica Flash F60 (60Å, 40-63um 230-400 Mesh) silica gel using forced flow by air (0.5-1.0 bar). ^{1}H and ^{13}C NMR spectra were recorded on Bruker Neo400 (400.17 MHz for 1H, 100.6 MHz for 13C), UNI600 (600.1 MHz for ^{1}H, 150.9 MHz for ^{13}C) and UNI500 (500.4 MHz for 1 H, 125.8 MHz for 13C) spectrometers. Deuterated solvents ($CDCl_3$, DMSO-d_6) were purchased from Cambridge Isotope Laboratories, Inc. NMR data were analyzed using MestReNova software (Mestrelab Research). Mass spectra were recorded on a MALDI-TOF Bruker Daltonics Microflex LRF instrument using 1,8,9- trihydroxyanthracene (dithranol, 10 mg/1mL solution in THF) as a matrix in either positive- or negative-ion modes. Hydrogel samples were dried using a Labconco 7522800 lyophilizer. pH measurements were performed at 23°C using Orion 320 pH meter.

Optical spectra were recorded on a Perkin-Elmer 365 UV-Vis spectrophotometer. Steady-state fluorescence measurements were performed on spectrofluorometer (FS920, Edinburgh Instruments, UK), equipped with R2658P red-sensitive PMT (Hamamatsu). Quartz fluorometric cells (1 cm path length; Starna) were used in all optical experiments. Time-resolved phosphorescence measurements were performed using a custom-built fiber-optic phosphorometer constructed around a multichannel data acquisition board (USB NI-6361, National Instruments) operating at 1 MHz frequency. The excitation sources were light-emitting diodes (LEDs) (Ledengin, LZ1), and the detector was an avalanche photodiode (C12703; Hamamatsu). For CELI measurements, the detector was a gateable PMT (H11526-20-NF, Hamamatsu) combined with preamplifier unit (C9999, Hamamatsu). The instrument time constant was < 1 µs (FWHM).

The system for oxygen titrations has been described previously.[1-3] Briefly, a cylindrical vial was fitted with a silicon rubber stopper, through which an oxygen electrode (CK, 4 pA current at zero oxygen) and two stainless steel needles, serving as gas inlet and outlet, were inserted. The vial could hold approximately 10 ml of liquid, but it was slightly underfilled, so that a flow of gas (argon) could be passed over the solution surface. The vial was positioned inside a temperature-controlled chamber (±0.1°C), equipped with a magnetic stirrer, and the excitation and collection fibers of the phosphorometer were positioned at 90° against the vial inside the chamber. A piece of *Oxygel*, wrapped in a fine net-like cloth, was tied with a string to the electrode close to its tip. The electrode current was amplified and digitized, and the readings were recorded at 10 s intervals. Phosphorescence lifetimes were measured by the phosphorometer and logged into a file. As argon was passed over the surface of the stirred probe solution, gradually replacing oxygen and other dissolved gases, synchronous recordings of phosphorescence lifetimes and pO_2 provided the raw data for the subsequent Stern-Volmer analysis. Typically, ca 500-700 data points were recorded in each titration run.

Scanning Electron Microscopy (SEM) experiments were carried out in the CDB Microscopy Core facility at the Perelman School of Medicine, University of Pennsylvania, using a Quanta 250 FEG scanning electron microscope (FEI, Hillsboro, OR, USA), 10 kV accelerating voltage. Swollen *Oxygel* samples were gradually frozen using a mixture of ethanol and dry ice, avoiding structural damage, and lyophilized. The samples were mounted on stubs and sputter-coated with gold-palladium.

2. Synthesis

BTAGluO^{t}Bu. To a solution of 1,3,5-benzenetricarboxylic acid (0.119 g, 0.56 mmol) in dry DMF (N,N-dimethylformamide, 5 mL), HBTU (1.25 g, 3.38 mmol) was added, and the reaction mixture was stirred for 10 min at r.t. DIPEA (1.1 ml, 8.45 mmol) was added to the reaction mixture in one portion, followed by addition of a solution of L-glutamic acid di-*t*-butyl ester hydrochloride (Glu(O^{t}Bu)$_2$·HCl, 1.00 g, 3.38 mmol) in dry DMF (5 mL). The mixture was stirred for 2 days at r.t., after which MALDI-TOF analysis confirmed that the reaction came to completion. The reaction mixture was poured into ice-cold HCl aq (0.01M), which resulted in formation of a white precipitate. The precipitate was isolated by centrifugation, washed with HCl aq (0.01M) and then dried in vacuum. The resulting solid was purified by column chromatography (silica gel, gradient: hexanes/EtOAc (70:30). Yield 0.45 g, 86%. MALDI-TOF (m/z): calc. for $C_{48}H_{75}N_3O_{15}$: 934.13, found 935.2 $[M+H]^+$. ^{1}H NMR ($CDCl_3$) δ, ppm: 8.43 (3H, S, -NH-), 7.16 (3H, d, J = 7.7 Hz, -Ph-), 4.70 (3H, td, J = 7.9, 4.7 Hz, -CH-), 2.48 – 2.32 (m, 6H, $-CH_2$-

), 2.32 – 2.19 (m, 3H, $-CH_2-$), 2.07 – 2.04 (m, 3H, $-CH_2-$), 1.50 (27H, s, $-(CH_3)_3$), 1.43 (27H, s, $-(CH_3)_3$). ^{13}C NMR (CDCl3) δ 172.2, 171.2, 165.1, 134.7, 128.57, 82.6, 80.77, 80.75, 52.9, 31.7, 28.1, 27.6.

BTAGluOH. BTAGluO^{t}Bu (0.45 g, 0.48 mmol) was dissolved in 1:1 mixture of dichloromethane (6 ml) and trifluoroacetic acid (6 ml). The solution was kept for 2h at r.t, after which the solvents were removed by rotary evaporation, and the target compound was dried in vacuum. Yield: 0.26 g, 92%. MALDI-TOF (m/z): calc. for $C_{24}H_{27}N_3O_{15}$: 597.49, found 598.50$[M+H]^+$. ^{1}H NMR (DMSO-d6), δδ, ppm: 8.97 (3H, d, J = 7.6 Hz, -NH-), 8.49 (3H, s, -Ph-), 4.46 (3H, ddd, J = 9.7, 7.6, 4.9 Hz, -CH-), 2.39 (6H, t, J = 7.5 Hz, $-CH_2-$), 2.16-1.98 (6H, m, $-CH_2-$). ^{13}C NMR (DMSO-d6) δ 174.27, 173.68, 166.39, 134.93, 129.73, 52.58, 30.84, 26.35.

***Oxygel* synthesis** (see Table S1 for compositions). BTAGluOH, PtTBP-AG^{m}OH (m=0-2) and HBTU (3.0 mg, 0.11 mmol) were dissolved in dry DMF (75 µL) and mixed for 10 min. NH_2-PEG-NH_2 (Av. MW 5000) in DMF (75 µL) was added to the mixture under rigorous agitation (shaking), followed by addition of DIPEA (1.4 µl, 0.11 mmol), which caused instant gelation. The resulting gel was kept in distilled water for 24h to remove impurities and by-products, then in HCl aq (0.1M) for 24h to extract unreacted PEG-diamine and then washed with distilled water several times.

Table S1. Compositions of the synthesized *Oxygels* PtTBP-OG^m (m=0-2).[a]

#	*M*	*N* [b]	PtTBP-AG^{m}OH nmol / µg	BTAGluOH nmol / µg	$PEG(NH_2)_2$ nmol / mg	N_{CO2H}/N_{NH2} [c]	f_p, % [d]	f_{H2O}, % [e]	*SR*, [f]
1	0	8	27.5 / 35	640 / 382	2000 / 10.0	1.015	0.36	98.4	54
2	1	16	27.5 / 105	640 / 382	2000 / 10.0	1.07	0.99	97.9	38
3	2	32	27.5 / 201	640 / 382	2000 / 10.0	1.18	1.90	95.3	23
4o	2	32	25.6 / 183	596 / 356	2000 / 10.0	1.10	0.98	95.3	21
5	2	32	12.0 / 88	602 / 360	2000 / 10.0	1.00	0.84	95.4	20
6	2	32	6.3 / 46	633 / 378	2000 / 10.0	1.00	0.44	96.0	24

[a] All *Oxygels* were synthesized using the general procedure outlined above. [b] Number of carboxylic acid groups in PtTBP-AG^{m}OH dendrimers; [c] Ratio between carboxylic acid and amino groups. [d] Fraction of PtTBP-AG^{m}OH dendrimer (by weight) in dry *Oxygels*; [e] Fraction of water (by weight) in

swollen *Oxygels*. [f] Swelling ratio in water at equilibrium. The error in determination of the *SR* was estimated to be ±5 based on 3 independent measurements.

3. *Oxygel* swelling

The swelling kinetics was investigated using the gravimetric method. A dried hydrogel sample was placed into deionized water. At selected time intervals it was removed, placed on a filter paper to remove residual water from its surface, after which its weight was measured on a microbalance. The swelling ratio (SR) was defined as follows:

$$SR = \frac{(weight\ of\ swollen\ sample - weight\ of\ dry\ sample)}{weight\ of\ dry\ sample}$$

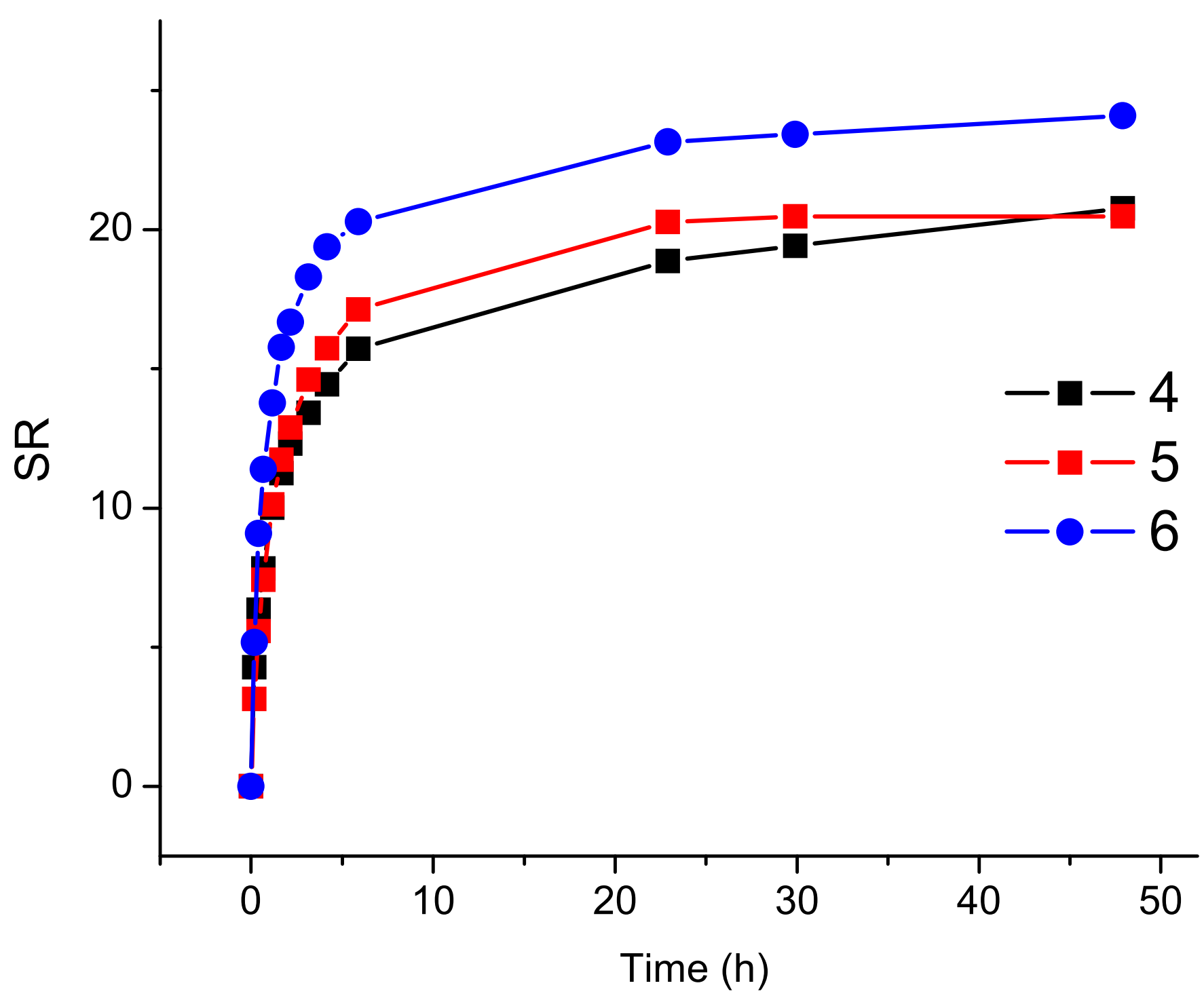

Figure S1. Swelling kinetics of *Oxygels* #4-6 (Table S1).

4. Optical absorption and emission spectra of *Oxygels*

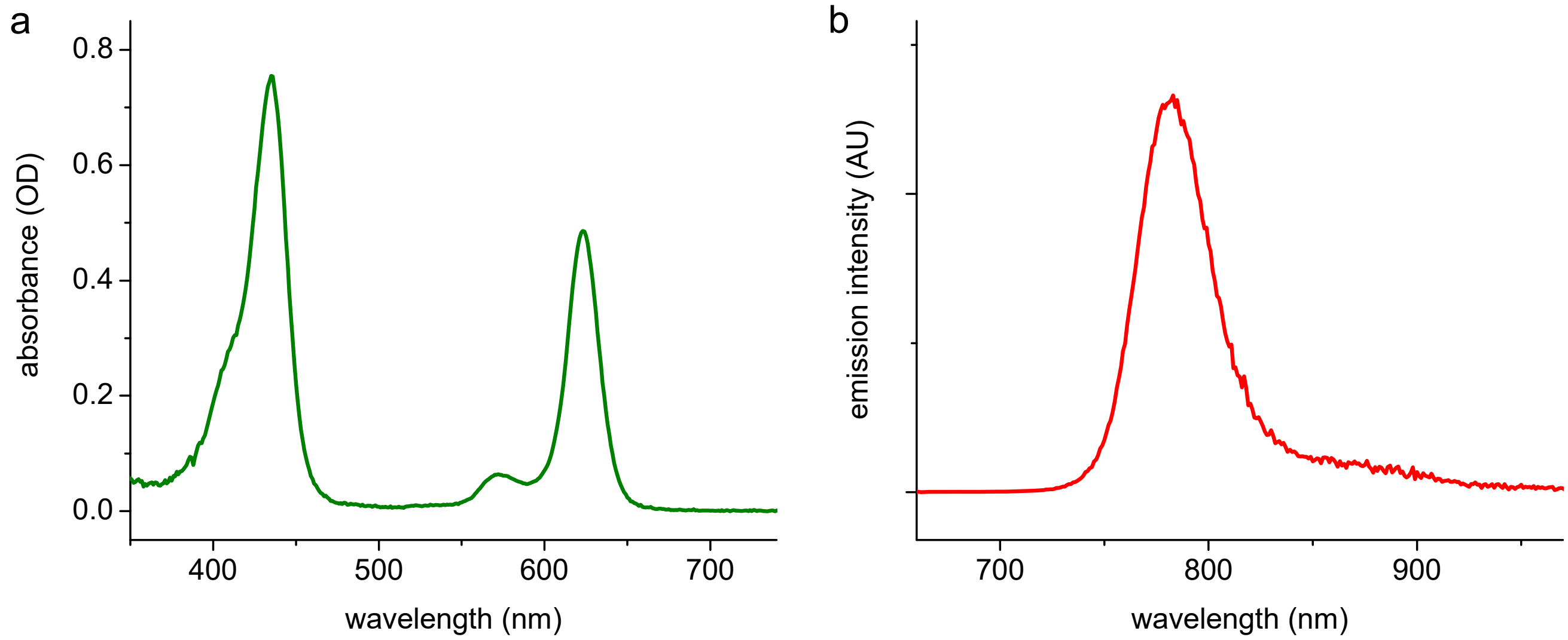

Figure S2. Electronic absorption (a) and phosphorescence (b) spectra of PtTBP-OG[2]. The spectra were recorded using a piece of hydrogel immersed in distilled water inside a quartz cuvette (2 mm optical path). For recording the phosphorescence spectrum (λ_{ex}=620 nm), the sample was deoxygenated by argon bubbling.

5. Structures of Pt porphyrin-dendrimers PtP-AG*m*PEG (*m*=0-2) [1]

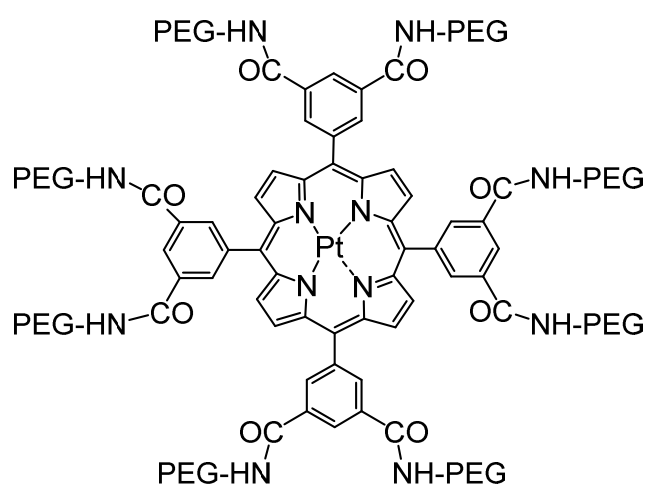

PtP-AG^{0}-PEG

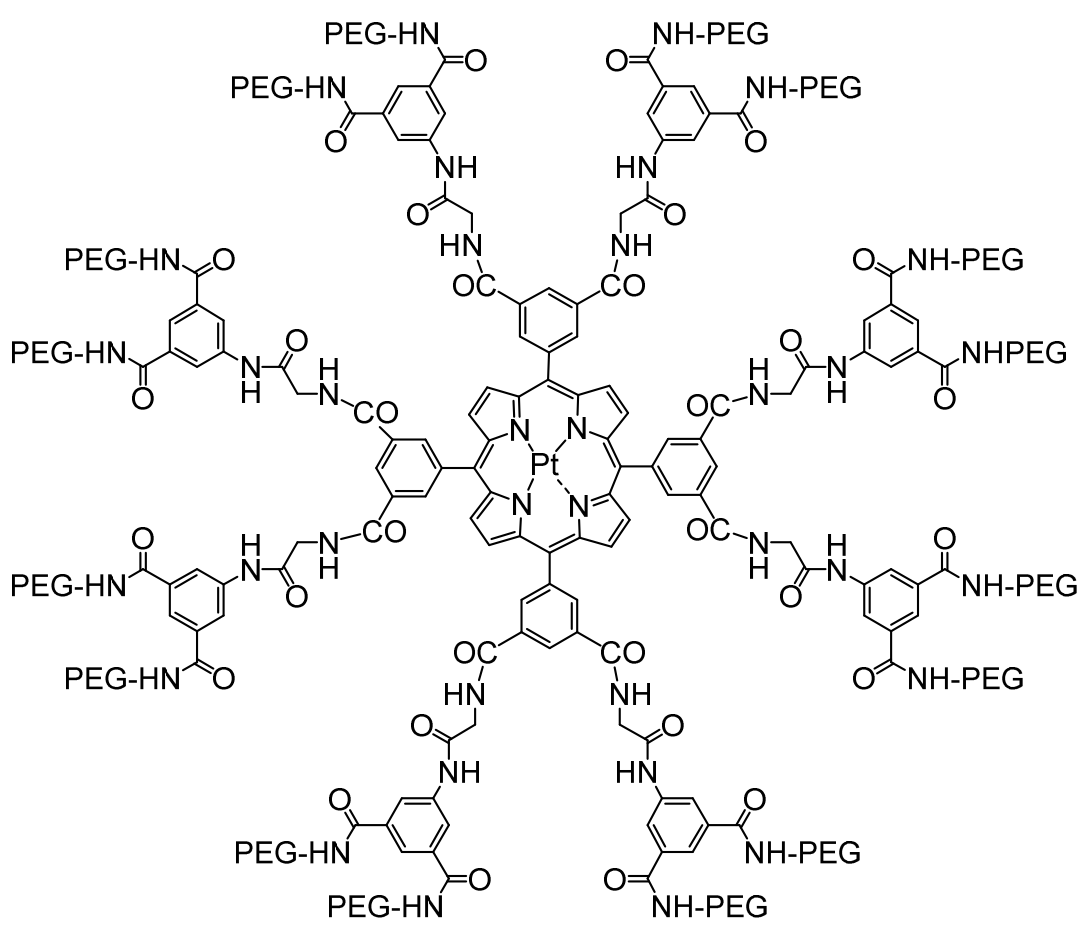

PtP-AG^{1}-PEG

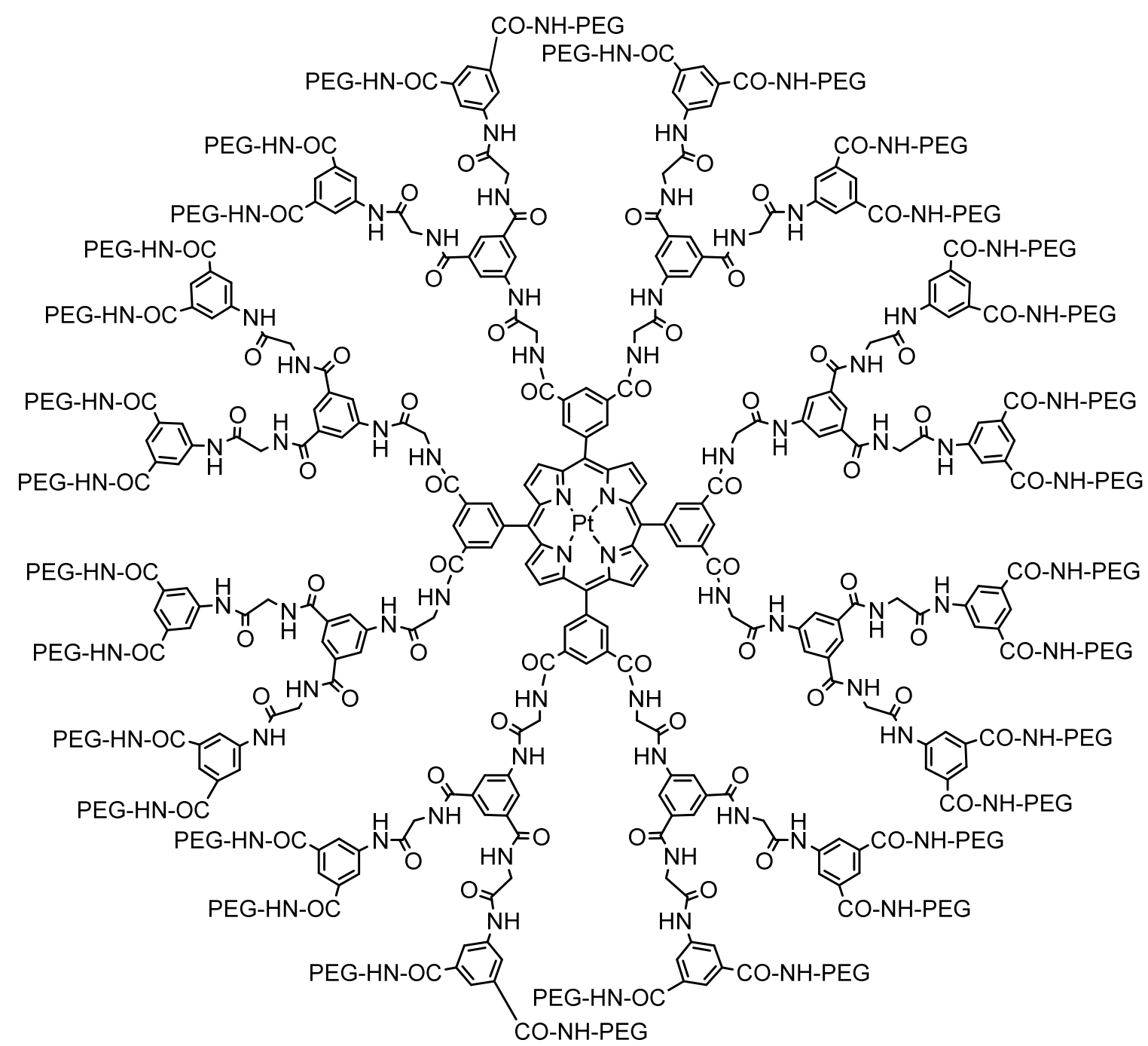

PtP-AG^{2}-PEG

PEG = MeO-(CH_2-CH_2-O)n-CH_2-   Av. MW 1000

6. Oxygen quenching plots

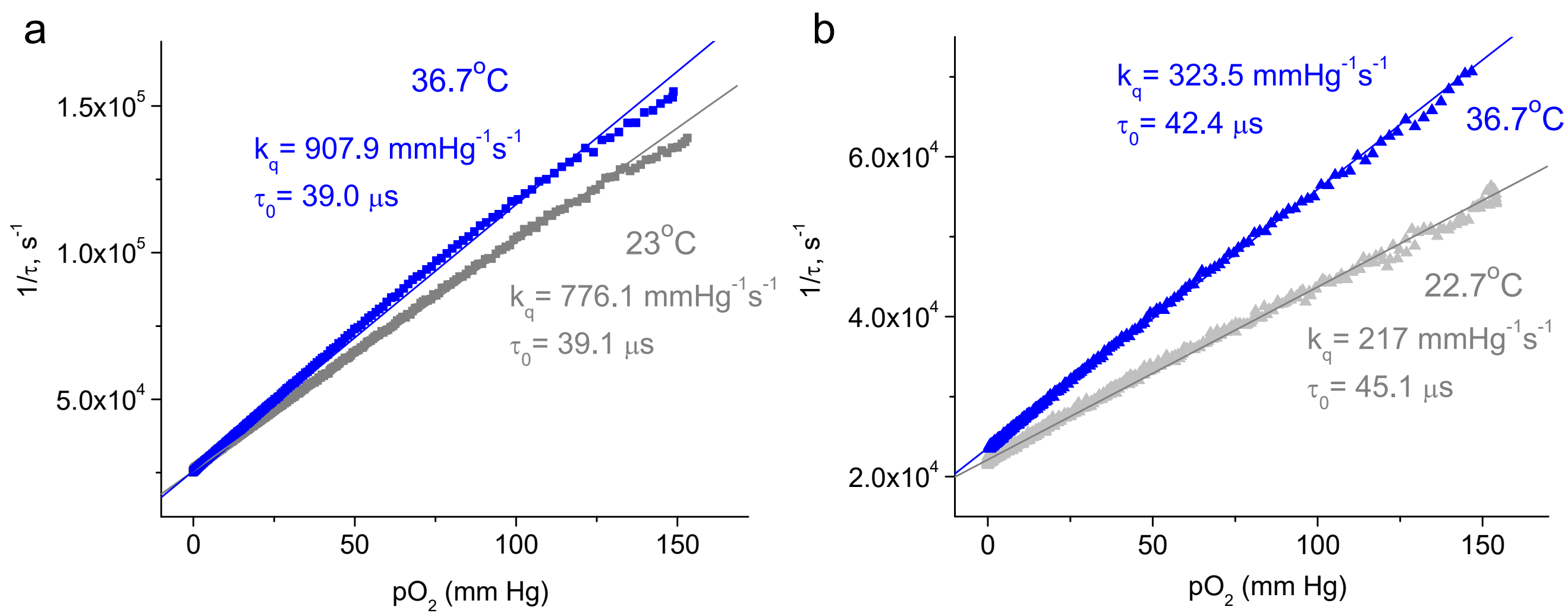

Figure S3. Stern-Volmer oxygen quenching plots of *Oxygels* PtTBP-OG[1] and *Oxygels* PtTBP-OG[2] at 37°C in phosphate buffer (20 mM, pH 7.2). The data for 23°C (as a reference) are shown in gray. 1/τ is the phosphorescence rate constant, where τ is the phosphorescence decay time. pO_2 is partial pressure of oxygen.

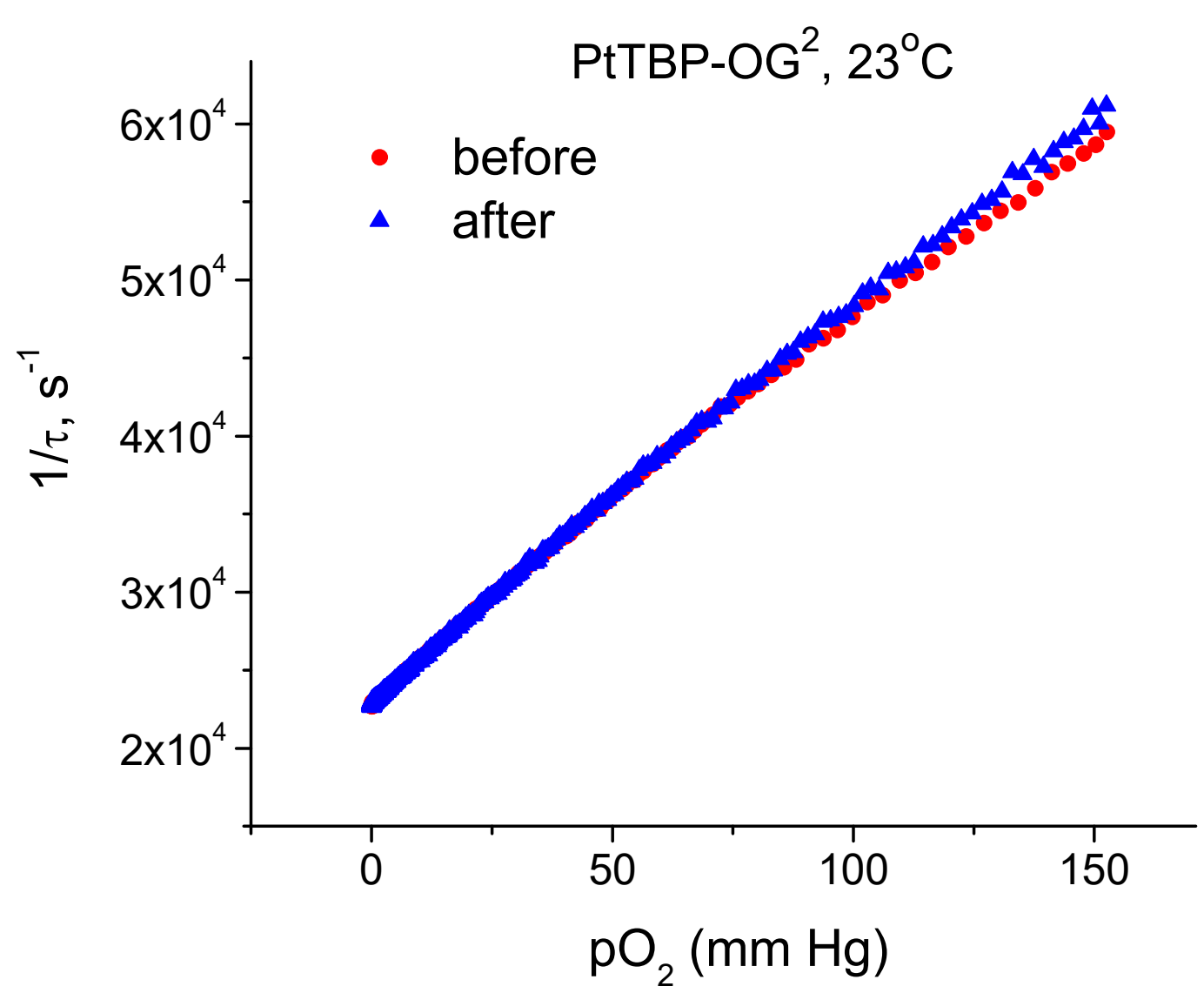

Figure S3a. Stern-Volmer oxygen quenching plots of *Oxygel* PtTBP-OG[2] at 23°C before and after desiccation and rehydration. 1/τ is phosphorescence rate constant, where τ is the phosphorescence decay time. pO_2 is partial pressure of oxygen.

7. *Oxygel* biocompatibility and biodegradability studies

Subcutaneous implantation of PtTBP-OG² and in vivo oxygen measurements. All animal procedures were conducted in accordance with the University of Pennsylvania IACUC regulations (protocol no: 806482).

16-20 weeks-old male mice (C57BL/6J, Jackson Laboratory) were housed in pairs in standard plastic ages filled with wooden chips in a conventional (semi-barrier) animal facility. Cages were enriched with Shepard shacks (Shepard Specialty Papers), clear plastic tunnels (Braintree Scientific), swings (Datesand Group) and two different kinds of nesting material (Nestlet, Enviro-dri; both Shepard Specialty Papers). Food and water were available *ad libitum*, and the light/dark cycle was 12/12h with lights on at 7 am and off at 7 pm.

For anesthesia induction mice were transferred into a Plexiglas box filled with 2.0% isoflurane (Dechra Veterinary Products) in pure oxygen (Vet Flo Isoflurane Vaporizer, Kent Scientific). During preparation and surgery mice were kept on a heating pad (37°C) to prevent hypothermia, and the anesthesia was maintained as 1.5% isoflurane/O_2 using a nose cone. Preparation included application of eye ointment, subcutaneous (s.c.) injection of Ethiqa XR (1 mg/kg; buprenorphine extended-release injectable suspension; Fidelis Animal Health) and shaving and disinfection (ethanol and povidone–iodine, Braunoderm, B. Braun) of the neck area.

PtTBP-OG^2 was cut into uniform pieces ~3-5 mm in diameter, ~3 mm-thick. The pieces were incubated in ethanol for 6-12h for sterilization and extensively washed with sterile phosphate buffer saline (PBS) solution prior to implantation.

Two small skin incisions (<10 mm) were made in the neck area, on the right and left sides of the neck, and subcutaneous cavities were created. One *Oxygel* implant was placed in each cavity, and the skin was closed with a single suture. A triple antibiotic ointment was applied locally in the surgery area, and the mice received s.c. injections of saline (0.5 ml) before being transferred into their home cages. The recovery was under infrared irradiation, and it was monitored closely. After appropriate recovery time, cages were transferred back to the holding room, and all mice were provided with Diet Gel (Clear H2O) and normal food.

To perform oxygen measurements, mice were anesthetized using isoflurane 1.5%/O_2, as described above, and the implantation side was shaved. Oxygen levels were measured at the location of the implant using a fiber-optic phosphorometer.

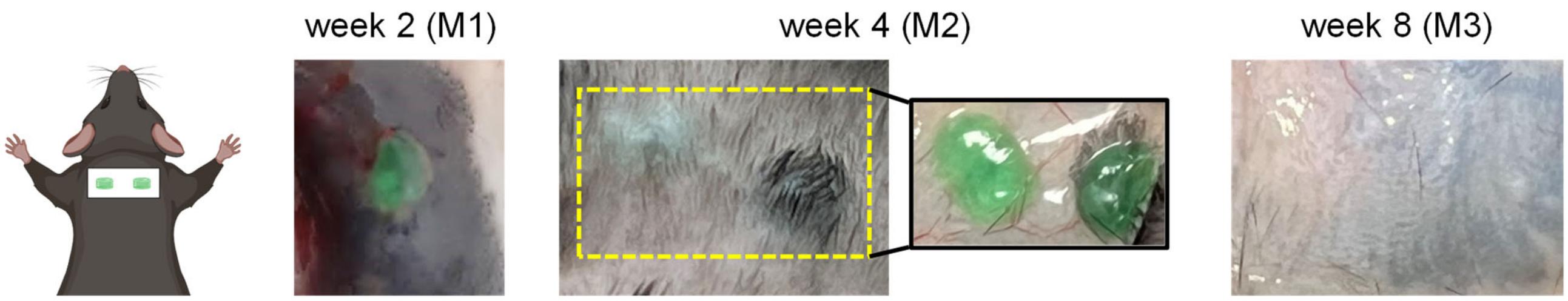

Figure S4. Subcutaneously implanted *Oxygels*. 'week 2' - the implant is adhered to the s.c. layer; its size is close to the original. 'week 4' - (left) prior to lifting the skin, the implants are well-seen under the fur; (right) the implants are adhered to the s.c. layer; they appear swollen, their size ~1.5 times larger than the original. 'week 8' - the area where the implants were expected to be localized.

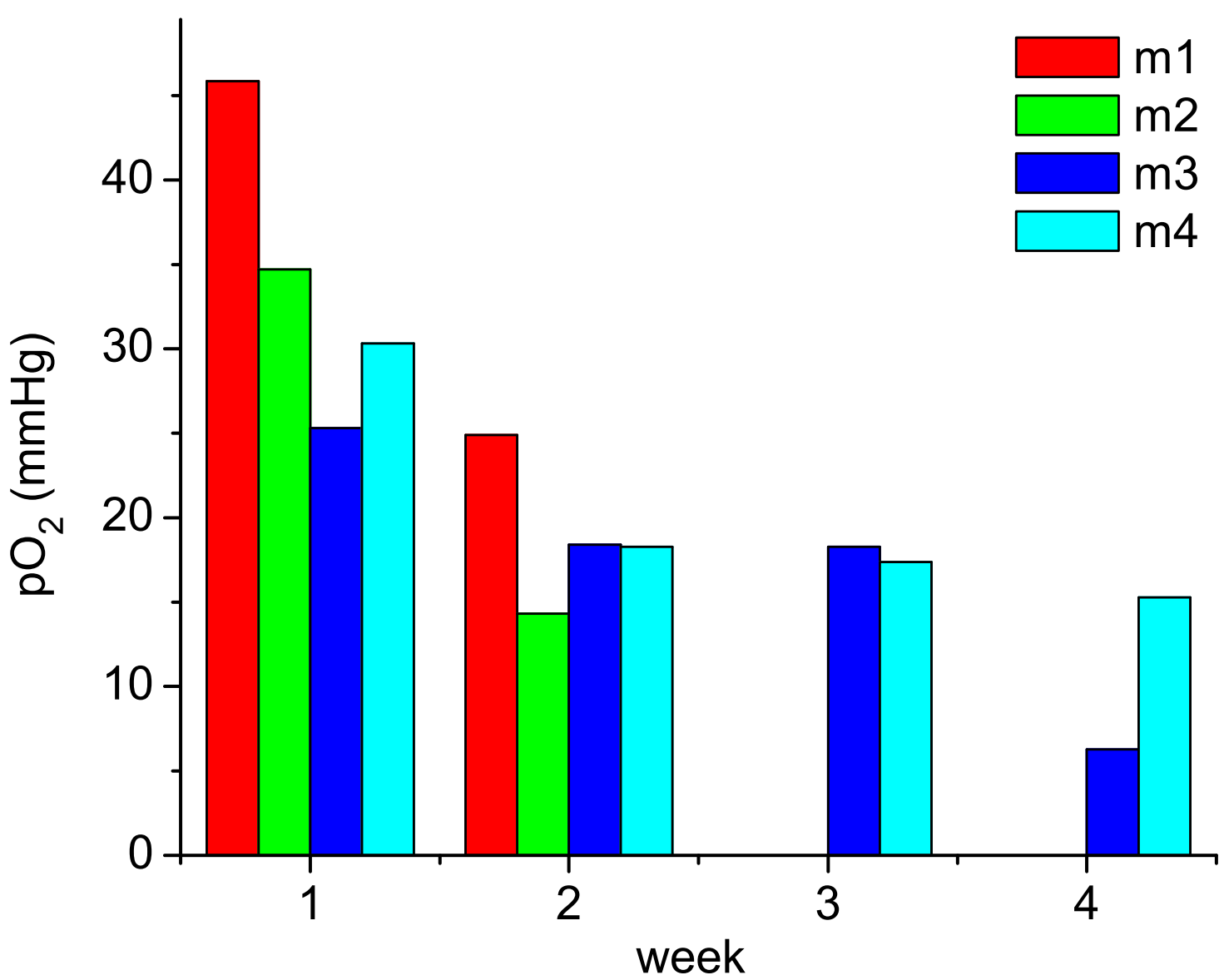

Figure S5. pO_2 values in mice (m1-m4) measured over the course of 4 weeks.

A total of 6 mice were used in two separate experiments (*Experiment 1*: n=4; *Experiment 2*: n=2). In *Experiment 1*, 4 mice underwent s.c. implantation surgery, after which oxygen measurements were performed every week. Mice were euthanized 2 weeks (n=2) or 4 weeks (n=2) after surgery by carbon dioxide overdose and cervical dislocation. Skin samples including remnants of *Oxygel* implants were dissected and fixed in 4% paraformaldehyde (Electron Microscopy Sciences) for 6h before sucrose treatment (30%) and cryo-embedding. In *Experiment 2*, 2 mice underwent implantation surgery, after which oxygen measurements were performed weekly starting 4 weeks after the surgery until euthanasia

at 8 weeks. Visual post-mortem examination of the s.c. area was performed to evaluate the presence of *Oxygel* residues (Fig. S4).

H&E and immunofluorescence staining. Histological sections (10 µm-thick) were prepared using cryo-tapes (Sectionlab) in a cryostat (Thermo Scientific) and stored at -20°C. For Hematoxylin and Eosin (H&E) staining, the sections were rehydrated in PBS, rinsed with tap water, and stained for 2 min with Harris Hematoxylin solution (Fisher Scientific). Subsequently, the sections were washed for 10 min with tap water and stained for 4 min with 0.5% Aqueous Eosin Y (Sigma Aldrich), then rinsed with tap water and mounted on cover slips using fructose solution (30% in distilled water; Sigma Aldrich).

For immunofluorescence staining of vessels (endomucin – EMCN) or of macrophages (F4/80), the sections were rehydrated in PBS, blocked with 10% goat serum for 30 min and incubated with either rat monoclonal EMCN antibody (1:100; clone: V.7C7; sc-65495; Santa Cruz) or rat monoclonal F4/80 antibody (1:400; clone: CI-A3-1; NB600-404; Novus Biologicals) in a staining buffer (PBS/5% FCS/0.1% Tween20 or PBS/5% goat/0.1% Tween20, respectively) for 2h at room temperature. The sections were washed with PBS/0.1% Tween20 and incubated with either goat anti-rat AF488 secondary antibody (A-11006; Thermo Fisher Scientific) or goat anti-rat AF647 secondary antibody (A-21247; Thermo Fisher Scientific) in staining buffer for 1.5h at room temperature. Finally, the sections were washed with PBS/0.1% Tween20, stained with DAPI (NucBlue Fixed Cell ReadyProbes Reagent; Invitrogen) and mounted on cover slips using Fluoromount-G (Invitrogen). Imaging was performed using a Axioscan 7 Microscope Slide Scanner (ZEISS Group).

8. Cherenkov-Excited Luminescence Imaging (CELI)

All CELI experiments were performed at the Department of Medical Physics, University of Wisconsin-Madison.

Animal procedures were approved by the University of Wisconsin School of Medicine and Public Health Institutional Animal Care and Use Committee (Protocol M006554-A03). Nude male mice 6 to 8 weeks of age (Envigo, IN, USA) were used in this study. The animals were initially anesthetized with 2.5 to 3% isoflurane delivered with air flow and maintained throughout the experiment under 2% isofluorane anesthesia. For both skin and under-intestine gel implantation, small incisions were made, and *Oxygel* implants were carefully placed at target depths. For subcutaneous implantation, the *Oxygel* PtTBP-OG^2 piece (~20 mg) was implanted at a depth of ~1 mm under the surface (Fig. 4, main text).

For intestinal implantation, a larger piece (~50 mg) was implanted under intestines ~3 mm under the surface. Vetbond was used to close the incisions.

Cherenkov light was induced by irradiation from a linear accelerator (linac; Mobetron, Intraop, USA) operating in FLASH mode (9 MeV, pulse width at half maximum 4 μs, 10 Hz pulse repetition rate). The imaging system comprised a gated intensified CCD camera (ICCD, PI-MAX4 1024i; Princeton Instruments), a lens (Canon Zoom Lens EF-S 55-250mm), and a 700 nm long-pass filter (Edmund Optics, USA). The camera was positioned ~1 m away from the linac and shielded by lead blocks from scattered X-ray photons.

For camera-based imaging, the dose was 0.25Gy per radiation pulse. The camera gate (~2 ns rise time) was synchronized with linac pulses, such that integration of phosphorescence on the CCD began at a pre-selected delay relative to the pulse and continued for 1 ms, resulting in a phosphorescence intensity image. Gating enabled suppression of the bright Cherenkov flash, which could overwhelm the detector and was not a part of the signal of interest. Typically, a phosphorescence intensity image was collected using 5 μs delay.

In addition, using a sequence of images with different delay times and subsequent pixel-wise fitting with exponentials, a phosphorescence lifetime image could be generated and subsequently converted to an image of the partial pressures of oxygen (pO_2 image). In that mode, after the initial delay of 5 μs, image acquisition was turned on with delays incremented in 2 μs steps, ending with a delay of 83 μs. Each image was integrated for 1 ms. In total, 40 phosphorescence intensity images were collected, corresponding to the total dose of 10Gy.

The functional phosphorescence lifetime information used for oxygen quantification was obtained from signals measured by a single-detector phosphorometer (Oxyled).[1, 2, 4] The phosphorometer was positioned ~1.5 m away from the linac, and its detector (avalanche photodiode, APD) was coupled to the object by a single-mode fiber (4 mm in diameter). Data acquisition was synchronized with linac pulses, whereby phosphorescence decays were collected upon excitation by a single pulse (7.9 Gy/pulse). The APD current was digitized at 500 kHz frequency. In addition, using a light emitting diode (630 nm) and another fiber, phosphorescence could be excited by light pulses.

When performing CELI of phosphorescence imaging with LED excitation, room lights were turned off to reduce noise.

References

1. A. Y. Lebedev, A. V. Cheprakov, S. Sakadzić, et al., "Dendritic phosphorescent probes for oxygen imaging in biological systems", *ACS Appl Mater Interfaces* 1 (2009): 1292. 10.1021/am9001698.

2. T. V. Esipova, A. Karagodov, J. Miller, et al., "Two new "protected" oxyphors for biological oximetry: properties and application in tumor imaging", *Anal. Chem.* 83 (2011): 8756. DOI: 10.1021/ac2022234.

3. V. Rozhkov, D. Wilson, S. Vinogradov, "Phosphorescent Pd porphyrin-dendrimers: Tuning core accessibility by varying the hydrophobicity of the dendritic matrix", *Macromolecules* 35 (2002): 1991.

4. T. Mani, D. M. Niedzwiedzki, S. A. Vinogradov, "Generation of phosphorescent triplet states *via* photoinduced electron transfer: energy and electron transfer dynamics in Pt porphyrin-rhodamine B dyads", *J. Phys. Chem. A.* 116 (2012): 3598−3610.

NMR spectra

^{1}HNMR , $CDCl_3$, BTAGlu[t]But

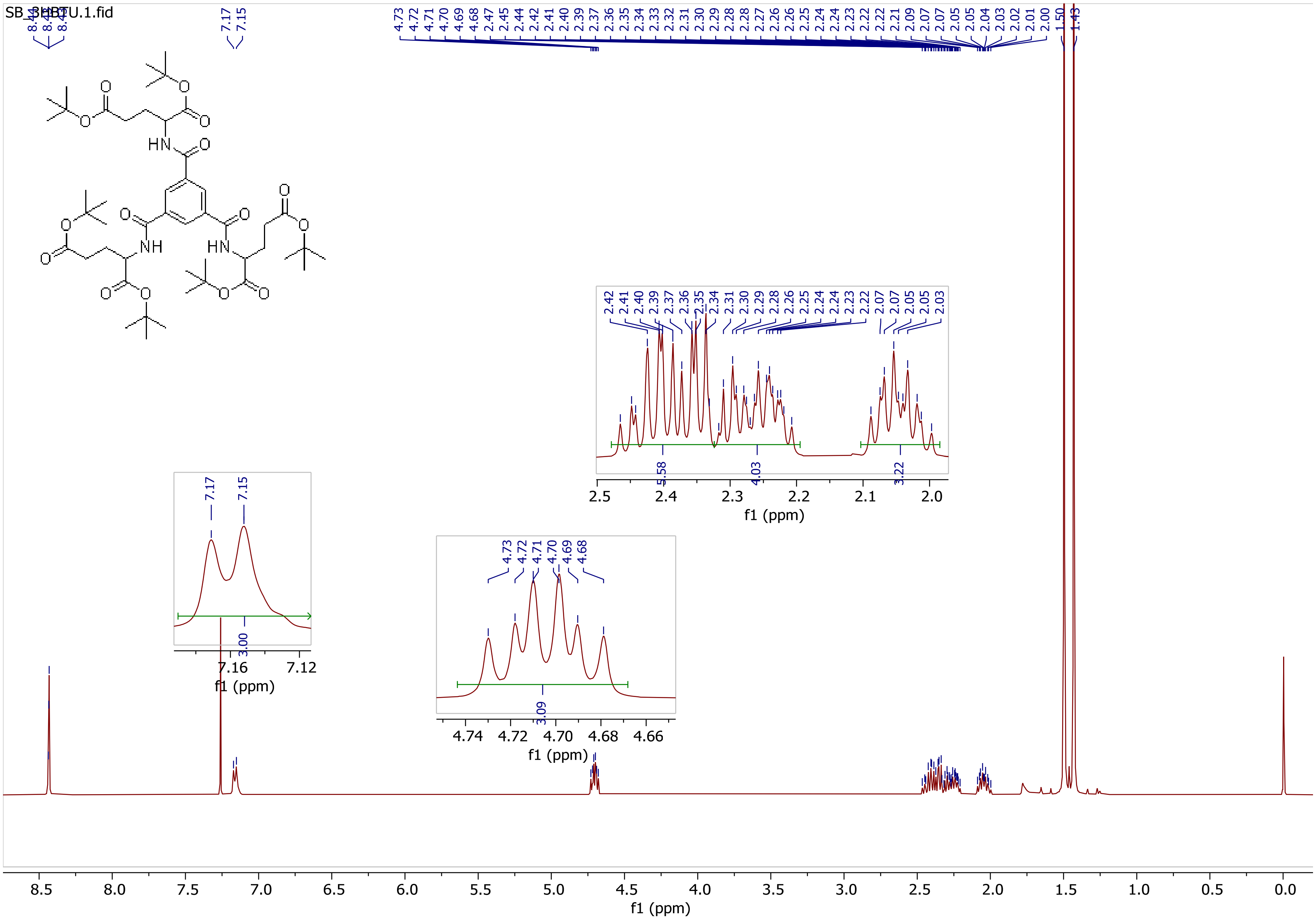

^{13}CNMR, $CDCl_3$, BTAGlu^{t}But

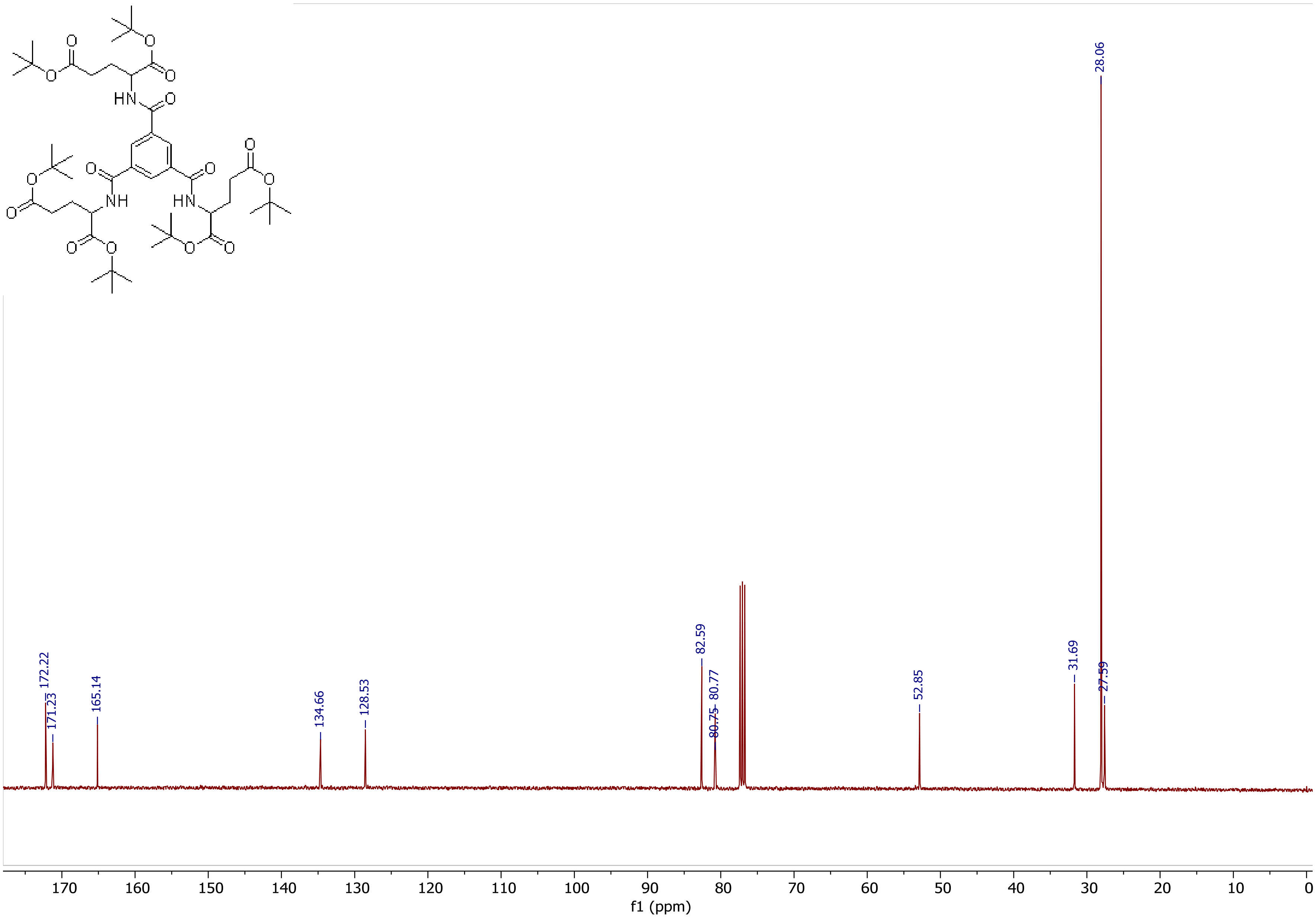

^{1}HNMR, DMSO-d6 , BTAGlu

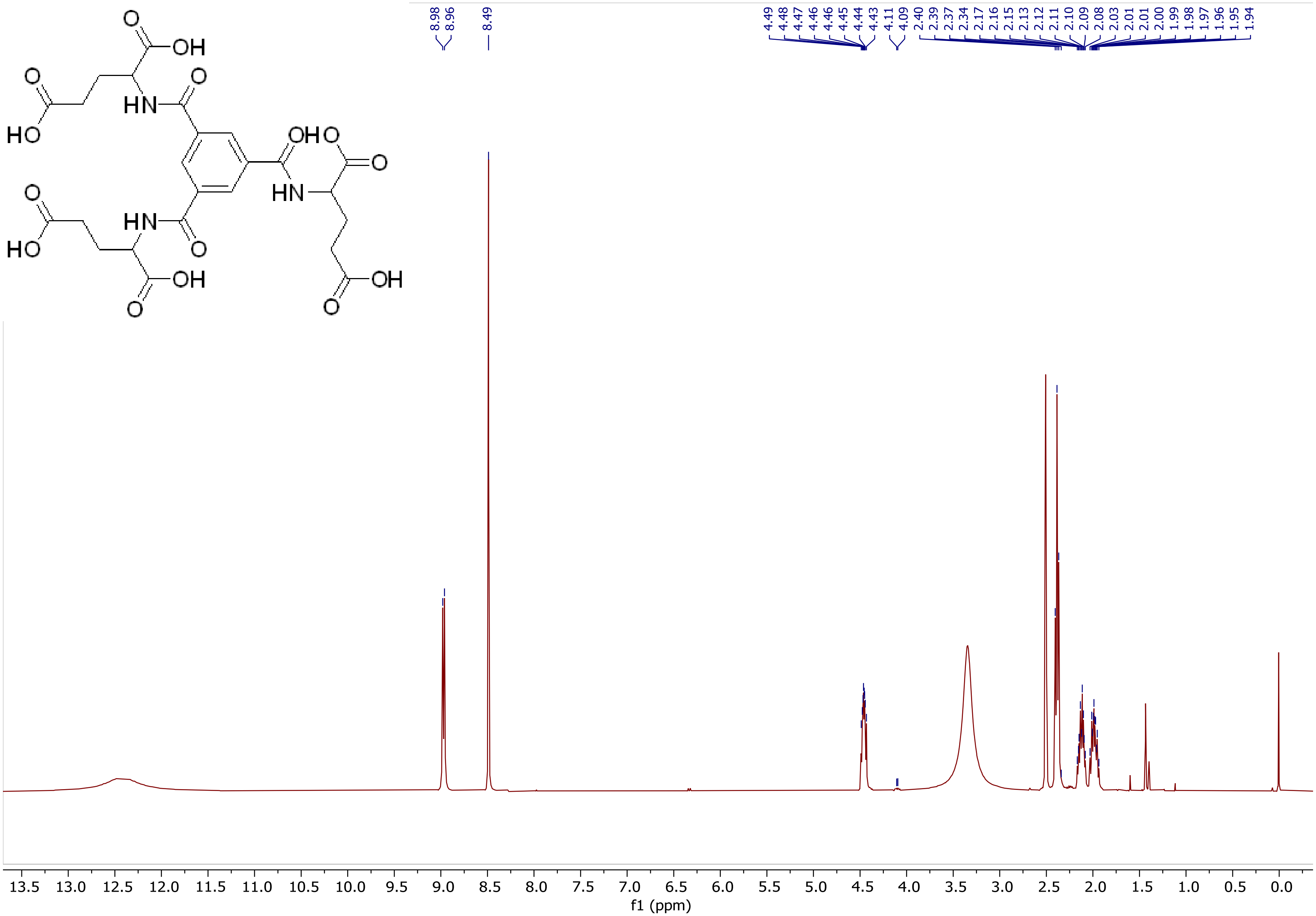

^{13}CNMR, DMSO-d_6 , BTAGlu

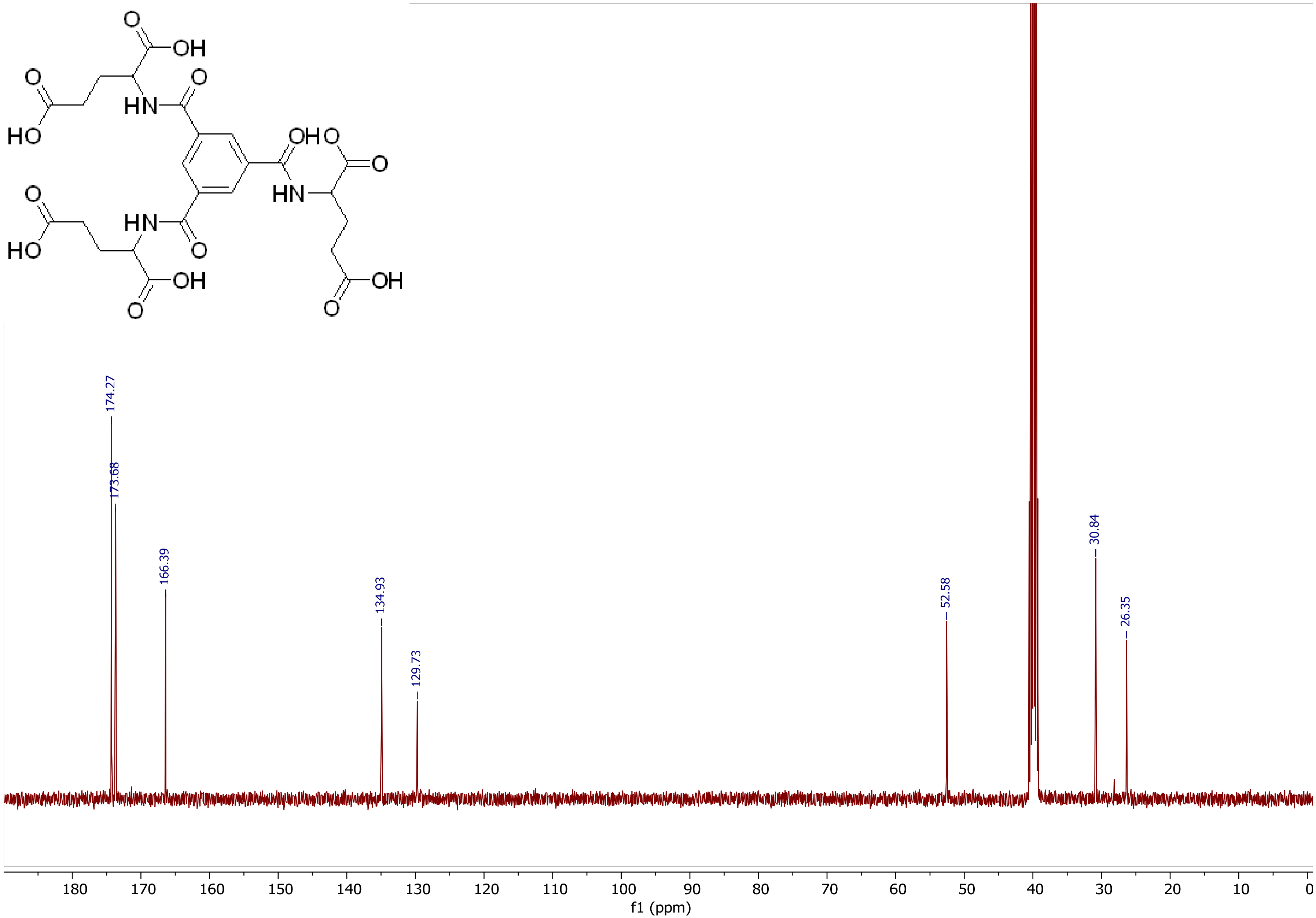

Supplement: Supplementary file 1 [file ADHM-15-0-s001.pdf]
